# Loss of p14 diminishes immunogenicity in melanoma via non‐canonical Wnt signaling by reducing the peptide surface density

**DOI:** 10.1002/1878-0261.13660

**Published:** 2024-05-28

**Authors:** Jonas Wohlfarth, Corinna Kosnopfel, Dominic Faber, Marion Berthold, Claudia Siedel, Melissa Bernhardt, Andreas Schlosser, Tyler Aprati, David Liu, David Schrama, Roland Houben, Dirk Schadendorf, Matthias Goebeler, Svenja Meierjohann, Bastian Schilling

**Affiliations:** ^1^ Department of Dermatology, Venereology and Allergology University Hospital Würzburg Germany; ^2^ Rudolf‐Virchow‐Centre for Integrative and Translational Bioimaging University of Würzburg Germany; ^3^ Dana‐Farber Cancer Institute Boston MA USA; ^4^ Harvard Medical School Cambridge MA USA; ^5^ Broad Institute of Harvard and MIT Cambridge MA USA; ^6^ Department of Dermatology, Comprehensive Cancer Center (Westdeutsches Tumorzentrum) German Cancer Consortium (DKTK, partner site Essen) and University Hospital Essen Germany; ^7^ Institute of Pathology University of Würzburg Germany

**Keywords:** CDKN2A, immunogenicity, immunotherapy, melanoma, peptide surface density, Wnt signaling

## Abstract

Immunotherapy has achieved tremendous success in melanoma. However, only around 50% of advanced melanoma patients benefit from immunotherapy. Cyclin‐dependent kinase inhibitor 2A (*CDKN2A*), encoding the two tumor‐suppressor proteins p14^ARF^ and p16^INK4a^, belongs to the most frequently inactivated gene loci in melanoma and leads to decreased T cell infiltration. While the role of p16^INK4a^ has been extensively investigated, knowledge about p14^ARF^ in melanoma is scarce. In this study, we elucidate the impact of reduced p14^ARF^ expression on melanoma immunogenicity. Knockdown of p14^ARF^ in melanoma cell lines diminished their recognition and killing by melanoma differentiation antigen (MDA)‐specific T cells. Resistance was caused by a reduction of the peptide surface density of presented MDAs. Immunopeptidomic analyses revealed that antigen presentation via human leukocyte antigen class I (HLA‐I) molecules was enhanced upon p14^ARF^ downregulation in general, but absolute and relative expression of cognate peptides was decreased. However, this phenotype is associated with a favorable outcome for melanoma patients. Limiting Wnt5a signaling reverted this phenotype, suggesting an involvement of non‐canonical Wnt signaling. Taken together, our data indicate a new mechanism limiting MDA‐specific T cell responses by decreasing both absolute and relative MDA‐peptide presentation in melanoma.

## Graphical abstract

Melanoma cells elude detection and killing by melanoma differentiation antigen (MDA)‐specific T cells via downregulation of p14. Reduction of p14 leads to a decrease of melanoma differentiation proteins via Wnt5a signaling and increases HLA class‐I expression in melanoma cells. Diminished MDA surface density prevents effective engagement of cognate T cells.

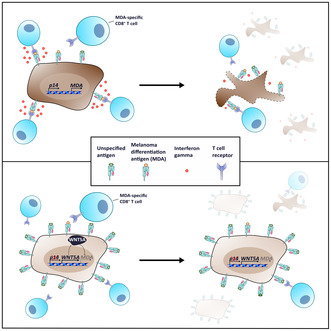

**Abbreviations**
ANOVA: analysis of variance
CDKN2A: cyclin‐dependent kinase inhibitor 2A
CM: complete medium
CTA or C/G: cancer/testis antigen
DCT: dopachrome tautomerase
Dox: doxycycline
EV: empty vector
Hilab: related to melanoma
HLA‐I: human leukocyte antigen class I
HRP: horse radish peroxidase
ICI: immune checkpoint inhibition
IFNg: interferon gamma
IL‐2: interleukin‐2
IQR: interquartile range
kd: knockdown
MDA or MD: melanoma differentiation antigen
MFI: mean fluorescence intensity
MHC: major histocompatibility complex
mOS: media overall survival
NR: non‐responder
OE: overexpressed in cancer
OS: overall survival
p14^ARF^
: p14
p16^INK4a^
: p16
pSILAC: pulsed stable isotope labeling by amino acids in cell culture
qPCR: quantitative real‐time PCR
R: responder
scr: scrambled
SD: standard deviation
shRNA: short hairpin RNA
siRNA: short interfering RNA
TCR: T cell receptor
TCR T cell: T cell receptor‐transgenic T cell
T_gp100_
: gp100‐specific TCR T cells
T_MART_
: MART‐1‐specific TCR T cells
TPM: transcripts per million

## 1 Introduction

Malignant melanoma is the deadliest type of skin cancer worldwide [1]. However, since the advent of immunotherapy, a substantial portion of metastatic melanoma patients achieve long‐term benefit and even survival [2, 3]. Despite advances in treatment regimens, around half of the advanced melanoma patients will not respond to anti‐PD‐1‐based immunotherapy [2]. Reasons for resistance to immunotherapeutic treatment are manifold and range from the lack of presented antigen on the tumor cell surface to genetic alterations [4, 5, 6, 7, 8].

One of the most frequently inactivated gene loci in metastatic melanoma is the tumor suppressor *CDKN2A* which encodes for the two gene products p14^ARF^ (p14) and p16^INK4a^ (p16) [9, 10, 11]. Approximately 45% of all melanoma cases show genetic alterations in the *CDKN2A* locus with mutations affecting p16 (43%) or p14 and p16 (57%) [12]. Additionally, transcriptional silencing by promoter hypermethylation poses a role in inactivating p14 and p16 and epigenetic repression can affect p14 and p16 independently of the other, diversifying the roles of p14 and p16. In 27% of the melanoma metastases, the p16 promoter was found to be hypermethylated, while around 57% of melanoma metastases showed promoter methylation of p14 [13].

Although p14 and p16 originate from the same gene locus, protein expression and functions differ as they are transcribed from alternative reading frames. While p16 regulates the cell cycle via inhibition of CDK4/6 and induces senescence [14, 15, 16], p14 prevents p53 from being ubiquitinated by MDM2, therefore driving p53 stability, apoptosis as well as senescence [17]. Studies in melanoma patients show a tendency toward a negative influence of *CDKN2A* inactivation on outcome of immune checkpoint inhibition (ICI) [16, 18, 19]. Accordingly, studies have shown that loss of *CDKN2A* is often accompanied by reduced CD8^+^ T cell infiltration in the tumor lesions [20, 21, 22]. Explanations for this circumstance focus largely on the role of p16 on immunogenicity of cancer cells and show that suppression of p16 may bypass senescence and decreases interleukin expression [23, 24]. In contrast, the influence of p14 on melanoma immunogenicity is insufficiently studied. Given the high frequency of *CDKN2A* alterations in melanoma and the increasing importance of ICI, it seems crucial to specifically investigate the resistance mechanisms driven by the suppression of p14.

An important prerequisite for successful ICI is the availability of antigens on cancer cells. In a physiological state, antigens are displayed as peptides on the surface of cells via HLA molecules to create immunological visibility [25]. During the evolution into a malignant tumor, melanoma cells change their immunopeptide surface landscape. Melanoma differentiation antigens (MDAs) such as gp100 or MART‐1 are expressed depending on the differentiation status of the melanoma cell [26, 27].

Another class of tumor‐specific antigens are cancer/testis antigens (CTAs) including PRAME, which was first discovered in melanoma and is mostly expressed in cancer and testis [28]. Both MDAs and CTAs are considered as good targets for melanoma‐specific therapeutic approaches as they are almost exclusively expressed on melanoma cells and are shared among patients. However, regardless of antigen origin, proper and sufficient presentation of antigens is crucial for T cell recognition.

As T cells are highly specific for their primed antigen, cancer cells manage to evade T cell attacks by abrogating the presentation of peptides on HLA molecules. For example, p53 is described to be involved in the adaptive immune response as it was found to upregulate HLA class‐I expression via TAP1 and peptide‐trimming via ERAP1 [29]. As p53 is an important downstream target, this suggests a role of the stress‐induced p14 signaling cascade in tumor immunogenicity.

Since downregulation of antigen presentation by cancer cells can have detrimental impact on T cell recognition and most studies do not differ between p14 and p16 or focus on p16 alone when analyzing *CDKN2A*, we wanted to explore the impact of p14 on melanoma immunogenicity. To this end, we generated p14 knockdown cell lines and cocultured them with melanoma‐specific T cells. In addition, we investigated the changes in surface HLA‐peptide distribution after p14 knockdown by mass spectrometry and show a new mechanism of p14 mediated alterations of the surface immunopeptidome.

## 2 Materials and methods

### 2.1 Cell culture

Human melanoma cell lines Ma‐Mel‐51 (MaMel51, RRID:CVCL_A186; provided by D. Schadendorf), SK‐MEL‐28 (RRID:CVCL_0526; ATCC), and WM35 (RRID:CVCL_0580; provided by M. Herlin) and their respective derived knockdown or control cell lines (scrambled, p14^kd^, p16^kd^ or double^kd^) were grown adherently in tissue culture flasks at 37 °C, 5% CO_2_ (characteristics are found in Table S1). Complete medium (CM) used to culture these cells consisted of RPMI‐1640 (Sigma‐Aldrich, Taufkirchen, Germany) with 10% (v/v) FCS (Biochrom, Berlin, Germany) and 1% (v/v) penicillin/streptomycin (Sigma‐Aldrich).

Cells from the lymphoblastoid cell line T2 (RRID:CVCL_2211; ATCC) express unstable, “empty” HLA‐A*02:01 class‐I molecules on their surface what makes them suited for experiments determining T cell recognition of HLA class‐I antigens by peptide‐pulsing the antigen of interest [30]. T2 cells were grown in suspension in tissue culture flasks in RPMI‐1640 with 10% FCS at 37 °C with 5% CO_2_. Cell characteristics are found in Table S1.

T cell receptor‐transgenic T cells (TCR T cells) were kept in X‐Vivo 15 (Lonza, Basel, Switzerland) with 50 U·mL^−1^ interleukin‐2 (IL‐2) (Peprotech, Hamburg, Germany). T cells were kept at 37 °C and 5% CO_2_ and medium was changed every second day.

Cell lines were regularly tested for *Mycoplasma* contamination via PCR‐based kit (AppliChem, Darmstadt, Germany) and always found to be negative. Cell lines were authenticated via genetic fingerprinting and were used no longer than 30 passages upon thawing of the cryostock.

p14 and p16 knockdown in cell lines with inducible gene constructs was induced by application of 2 μg·mL^−1^ doxycycline (dox) (Sigma‐Aldrich) for six days with a renewal at day four.

Wnt5a inhibition was achieved by applying 100 μm Wnt5a inhibitor Box5 for 48 h (Selleckchem, Houston, TX, USA).

p53 stabilization by MDM2‐inhibition was carried out by applying 10 μm Nutlin‐3 for 24 h (Sigma‐Aldrich).

### 2.2 p14/p16 shRNA and gp100 expression constructs

The lentiviral vectors induc_shRNA_p14 and induc_shRNA_p16 allowing dox‐induced expression of shRNAs targeting p14 (Accession number: NM_058195.4) and p16 (Accession number: NM_000077.5), respectively were transduced into melanoma cell lines MaMel51 (p14, p16 and p14 + p16), SK‐MEL‐28 (p14, p16, and p14 + p16), and WM35 (p14). As a control, a scrambled (scr) shRNA targeting no human transcript was used. Sequences of shRNAs are listed in Table S2 and the parental inducible vector is deposited (gene bank accession number: MH749464).

Overexpression of PMEL17 (gp100) in MaMel51 cell lines was conducted by lentiviral transduction of the pCDH_PMEL17_BSD vector encoding gp100 (Accession number: NM_006928.4). Transduction of pCDH_BSD served as an empty vector control (EV). Lentiviral transduction was carried out according to methods described in [31].

### 2.3 siRNA transfection

siRNA for *CDKN2A* (Horizon, Cambridge, UK) and *WNT5A* (Invitrogen, Carlsbad, CA, USA) was transfected according to manufacturer's protocol for Lipofectamine RNAiMAX transfection reagent (Invitrogen). Cells were analyzed or used in cocultures 72 h after transfection. The sequences of siRNAs are found in Table S2.

### 2.4 Peptide pulsing of cells

For peptide‐pulsing of T2 target cells, cells were prepared by culturing them at 26 °C for 16 h prior to addition of gp100_154‐162_ (peptides & elephants, Henningsdorf, Germany) or MART‐1_27‐35_ (Proteogenix, Schiltigheim, France) peptides. Peptides were then added in RPMI‐1640 to melanoma or prepared T2 cells for 3 h at 37 °C. If not stated otherwise, final peptide concentration was 10 μg·mL^−1^. Cells were then used in cocultures or analyzed by flow cytometry. The sequences of peptides are found in Table S3.

### 2.5 Generation of TCR T cells and cocultures

Generation of cytotoxic HLA‐A*02‐restricted TCR T cells with T cell receptors targeting gp100_154‐162_ and MART‐1_27‐35_ was carried out as previously described [32]. Lentivirus was produced by transfection of HEK293T cells (RRID:CVCL_0063; ATCC). On day ten after lentiviral transduction, TCR T cells (either gp100‐specific T_gp100_ cells or MART‐1 specific T_MART_ cells) by coculturing them in a 1 : 1 ratio with melanoma target cells for 72 h in 24‐well plates or with T2 cells in polypropylene tubes for 24 h, respectively.

For HLA‐I blockade, melanoma cells were pre‐treated with 1 μg·mL^−1^ pan‐HLA‐I blocking antibody (W6/32; Biolegend; #311402, San Diego, CA, USA) before coculture.

### 2.6 ELISA

Interferon gamma (IFNg) from coculture supernatants was quantified with the ELISA MAX Deluxe Set Human IFNg Kit (BioLegend) according to the manufacturer's protocol.

### 2.7 Flow cytometry cell surface staining

Fluorophore‐coupled antibodies were used to analyze surface expression of CD25 (CD25‐APC; Biolegend; #302610) on TCR T cells and HLA‐A*02 (HLA‐A2‐APC; eBioscience/Thermo Fisher Scientific; #17‐9876‐42, Waltham, MA, USA), HLA‐A/B/C (HLA‐A,B,C‐PE; Biolegend; #311405), and PD‐L1 (CD274‐APC; Biolegend; #329708) expression on melanoma cells. Tetramer stainings of T cell receptors were performed with Flex‐T HLA‐A*02:01 monomer UVX (Biolegend; #280003) which were peptide‐loaded with gp100_154‐162_ or MART‐1_27‐35_ peptides and tetramerized according to manufacturer's protocol. Loaded tetramers were conjugated with streptavidin‐APC (Biolegend; #405207) and used in flow cytometry surface stainings to detect T cell receptors. Stainings were measured with a FACS Canto cytometer (BD, Franklin Lakes, NJ, USA). FACS data analyzes were performed with flowjo™ v10.8 software (BD Life Sciences, East Rutherford, NJ, USA).

### 2.8 Western blotting

Cell pellets were lysed with RIPA buffer (25 mm Tris HCl pH 7.6, 150 mm NaCl, 1% NP‐40, 1% sodium deoxycholate, 0.1% SDS) containing complete protease inhibitors (Roche, Basel, Switzerland) and PhosSTOP phosphatase inhibitors (Roche). Cell lysates were resuspended in 5x Pierce™ Lane Marker Reducing Sample buffer (Thermo Fisher Scientific) and loaded on a SDS‐PAGE with subsequent transfer to a 0.2 μm nitrocellulose membrane (Amersham, Amersham, UK). Blocking of membrane after transfer was done with 5% milk (Roth, Karlsruhe, Germany) in TBST (TBS with 0.05% Tween) for 1 h. Afterwards, the following primary antibodies were applied to the blocked membrane for 16 h: anti‐PMEL17 (Santa Cruz Biotechnology; #sc‐377 325, Heidelberg, Germany), anti‐MART‐1 (Agilent Technologies; #M7196, Glostrup, Denmark), anti‐p14ARF (Cell Signaling; #74560, Cambridge, UK), anti‐p16 (Proteintech; #10883‐1‐AP, Rosemont, IL, USA), anti‐p53 (Santa Cruz Biotechnology; #sc‐126), anti‐Wnt5a/b (Cell Signaling; #2530), anti‐Vinculin (Sigma‐Aldrich; #V9131‐100 μL), and anti‐GAPDH XP (Cell Signaling; #5174). Bands were detected after incubation with anti‐rabbit or anti‐mouse IgG horse radish peroxidase (HRP)‐conjugated secondary antibodies (Agilent Technologies; #P0448 or #P0260, Glostrup) for 1 h and chemiluminescence reaction with ECL solution (2.5 mm Luminol, 0.4 mm *p*‐coumaric acid, 0.018% H_2_O_2_ in 0.1 m Tris pH 8.5). Detection of bands was done with the Amersham Western Blot Imager 600 (Amersham).

### 2.9 Real‐time quantitative polymerase chain reaction (qPCR)

For differential transcriptional gene analysis, qPCR after RNA extraction and cDNA synthesis was conducted as previously described [32]. Primer sequences are listed in Table S2.

### 2.10 RNAseq analysis of melanoma cell lines

In‐depth *CDKN2A* knockdown analysis of MaMel51 scr/p14^kd^/p16^kd^ and double^kd^ cells was measured via bulk RNAseq. After RNA extraction with the Direct‐zol RNA Miniprep Kit (Zymo Research, Freiburg, Germany), RNA quality was checked using a 2100 Bioanalyzer with the RNA 6000 Nano kit (Agilent Technologies, Santa Clara, CA, USA). The RIN for all samples was ≥ 7.8. DNA libraries suitable for sequencing were prepared from 400 ng of total RNA, with oligo‐dT capture beads for poly‐A‐mRNA enrichment using the TruSeq Stranded mRNA Library Preparation Kit (Illumina, San Diego, CA, USA) according to manufacturer's instructions (½ volume). After 15 cycles of PCR amplification, the size distribution of the barcoded DNA libraries was estimated ~ 340 bp by electrophoresis on Agilent DNA 1000 Bioanalyzer microfluidic chips.

Sequencing of pooled libraries spiked with 1% PhiX control library, was performed at 25 million reads/sample in single‐end mode with 75 nt read length on the NextSeq 500 platform (Illumina) using 2x high output sequencing kit. Demultiplexed FASTQ files were generated with bcl2fastq2 v2.20.0.422 (Illumina).

To assure high sequence quality, Illumina reads were quality‐ and adapter‐trimmed via Cutadapt version 2.5 using a cutoff Phred score of 20 in NextSeq mode, and reads without any remaining bases were discarded (command line parameters: ‐‐nextseq‐trim = 20 ‐m 1 ‐a CTGTCTCTTATACACATCT) [33]. Processed reads were mapped to the human reference genome GCF_000001405.39_GRCh38.p13 with splice junction‐sensitive read mapper star version 2.7.2b using default parameters [34]. Read counts on exon level summarized for each gene were generated using featurecounts v1.6.4 from the subread package [35]. Multi‐mapping and multi‐overlapping reads were counted strand‐specific and reversely stranded with a fractional count for each alignment and overlapping feature (command line parameters: ‐s 2 ‐t exon ‐M ‐O ‐‐fraction).

The read abundance estimation and their differential expression were analyzed between axon and soma sample libraries using deseq2 version 1.24.0 [36]. Read counts were normalized by DESeq2 and fold‐change shrinkage was applied by setting the parameter “betaPrior = TRUE.” Genes identified with the adjusted *P*‐value < 0.05 and |log_2_FoldChange| ≥ 1 by using the Benjamini–Hochberg procedure were considered as significantly expressed between two samples. The enricher function was used to perform hypergeometric tests based on lists of significant genes, and the GSEA function was applied for gene set enrichment analysis considering the DESeq2 log_2_FoldChange of all analyzed genes. The RNAseq data are deposited at GEO (Accession number: GSE249763).

### 2.11 Analysis of patient samples

Bulk RNAseq data was downloaded from the Liu, Schilling, et al. study [37]. Data was downloaded on November 22^nd^, 2023, from these links: https://www.nature.com/articles/s41591‐019‐0654‐5#data‐availability and https://github.com/vanallenlab/schadendorf‐pd1. Patient samples were filtered to those with homozygous deletions of *CDKN2A* (*n* = 23). These patients were then stratified by response to PD‐1 immunotherapy. Response and non‐response definitions followed the source study. This led to 15 non‐responders and 8 responders across the samples. Between responders and non‐responders, transcripts per million (TPM) values for genes of interest were compared to achieve relative expression levels.

Survival analysis was conducted via the cBioPortal in *n* = 363 samples of cutaneous melanoma (TCGA, PanCancer Atlas study), accessed on December 12, 2023 [38]. Patients were stratified based on B2M and gp100 expression using the Onco Query Language (B2M: EXP < 0.05 [*n* = 231]; B2M: EXP > 0.05 [*n* = 132]; gp100: EXP > 0.1 [*n* = 114]; gp100: EXP < 0.1 [*n* = 249]). mRNA expression *z*‐scores relative to diploid samples (RNA Seq V2 RSEM) were used. Median OS (mOS) was calculated by the Kaplan–Meier Method.

Best radiographic response analysis was performed by using cBioPortal accessed on March 1, 2024 in *n* = 144 melanoma samples from the Liu and Schilling et al. [39] dataset obtained prior to anti‐PD‐1 ICI. Patients were stratified based on the presence (*n* = 32) or absence (*n* = 112) of a homozygous deletion of *CDKN2A*. Clinical annotation has been described previously.

### 2.12 Alamar blue viability assay

Viability of melanoma cells after coculture was analyzed by Alamar Blue assay. Briefly, resazurin was added to the medium to a final concentration of 0.017 mg·mL^−1^. Cells were incubated at 37 °C, 5% CO_2_ for 1 h. Afterward, 100 μL of the supernatant was transferred to a 96‐well plate, and fluorescence was measured at ex540 nm/em580 nm at the microplate reader Infinite M200 Pro (Tecan, Männedorf, Switzerland).

### 2.13 Pulsed stable isotope labeling by amino acids in cell culture (pSILAC), immunopeptidomics, and proteomics

Metabolic labeling by pulsed SILAC (pSILAC) was conducted prior to immunopeptidomic and proteomic analysis [40]. As described above, MaMel51 scr and p14^kd^ cells were treated with dox. Subsequently, dox‐treated cells were kept in RPMI SILAC medium supplemented with heavy‐labeled amino acids (Arg10, Lys8, Leu6) and dox, whereas untreated cells were kept in medium with medium‐heavy‐labeled amino acids (Arg6, Lys4, Leu3). After 24 or 48 h, respectively, the metabolically labeled cells were combined in a 1 : 1 ratio, and HLA‐I peptides were isolated by immunoaffinity purification and analyzed by nanoLC‐MS/MS as previously described with some minor modifications [41]. *De novo* sequencing was performed with PEAKS Xpro (Bioinformatics Solutions Inc., Waterloo, ON, Canada) including oxidation (Met), pyroglutamate formation on N‐terminal glutamine, carbamidomethylation (Cys) and the isotope labels for Arg10, Lys8, Leu6, and Arg6, Lys4, Leu3 as variable modifications. For every peptide a total of six variable modifications were allowed. The *de novo* results were exported and analyzed with Peptide‐PRISM [42] using the 6‐frame translated human genome (GRCh38) and the 3‐frame translated human transcriptome (ENSEMBL release 90). HLA peptide binding predictions were performed with NetMHCpan4.0 [43]. maxquant 2.0 [44] was used to determine H/M ratios for all HLA peptides identified by Peptide‐PRISM. Therefore, a custom fasta database containing all HLA peptides identified by Peptide‐PRISM from all four samples (scr_24h, scr_48h, p14^kd^_24h, p14^kd^_48h) at an FDR of 10% was generated. This database was used with MaxQuant with digestion mode set to “no digestion.” Multiplicity for the SILAC‐labeling was set to 3 (light, medium, and heavy) with a maximum of 6 labels per peptide. Arg6, Lys4, and Leu3 were selected as medium labels and Arg10, Lys8, and Leu6 as heavy labels. FDR filtering was turned off by setting PSM FDR, protein FDR, and site decoy fraction to 1. Minimum scores for modified and for unmodified peptides were set to 25. Finally, the re‐quantify option was used for improving quantification of large ratios. Apart from these adapted settings, the MaxQuant default parameters were used. The MaxQuant peptides table was merged with the Peptide‐PRISM results table by peptide sequence, and MaxQuant H/M ratios were used to generate scatter plots showing the modulation of the immunopeptidome.

For proteome analysis, cell lysates of combined pSILAC samples were precipitated with fourfold volume of acetone overnight at −20 °C. Pellets were washed with acetone at −20 °C. Precipitated proteins were dissolved in NuPAGE LDS sample buffer (Life Technologies, Carlsbad, CA, USA), reduced with 50 mm DTT at 70 °C for 10 min, and alkylated with 120 mm iodoacetamide at room temperature for 20 min. Separation was performed on NuPAGE Novex 4–12% Bis‐Tris gels (Life Technologies) with MOPS buffer according to manufacturer's instructions. A sample amount corresponding to 500 000 input cells was loaded on the gel. The gel was washed three times for 5 min with water and stained for 1 h with Simply Blue Safe Stain (Life Technologies). After washing with water for 1 h, each gel lane was cut into 15 slices.The excised gel bands were destained with 30% acetonitrile in 0.1 m NH4HCO3 (pH 8), shrunk with 100% acetonitrile, and dried in a vacuum concentrator (Concentrator 5301; Eppendorf, Hamburg, Germany). Digests were performed with 0.1 μg trypsin (Trypsin Gold, Mass Spectrometry Grade; Promega, Madison, WI, USA) per gel band overnight at 37 °C in 0.1 m NH_4_HCO_3_ (pH 8). After removing the supernatant, peptides were extracted from the gel slices with 5% formic acid, and extracted peptides were pooled with the supernatant.

NanoLC‐MS/MS analyses were performed on an Orbitrap Fusion (Thermo Scientific, Waltham, MA, USA) equipped with a PicoView Ion Source (New Objective, Littleton, MA, USA) and coupled to an EASY‐nLC 1000 (Thermo Scientific). Peptides were loaded on a trapping column (2 cm × 150 μm ID, PepSep; Bruker, Billerica, MA, USA) and separated on a capillary column (30 cm × 150 μm ID, PepSep; Bruker) both packed with 1.9 μm C18 ReproSil and separated with a 45 min linear gradient from 3% to 30% acetonitrile and 0.1% formic acid and a flow rate of 500 nL·min^−1^.

Both MS and MS/MS scans were acquired in the Orbitrap analyzer with a resolution of 60 000 for MS scans and 30 000 for MS/MS scans. HCD fragmentation with 35% normalized collision energy was applied. A top speed data‐dependent MS/MS method with a fixed cycle time of 3 s was used. Dynamic exclusion was applied with a repeat count of 1 and an exclusion duration of 30 s; singly charged precursors were excluded from selection. Minimum signal threshold for precursor selection was set to 50 000. Automatic gain control (AGC) was used with manufacturer's standard settings for MS scans and MS/MS scans. EASY‐IC was used for internal calibration. Raw MS data files were analyzed with maxquant version 2.0 [44]. The search was performed against the Ensemble—(June 4, 2021, UP000000589, 55 341 entries) and with tryptic cleavage specificity, allowing 3 miscleavages. Protein identification was under control of the false‐discovery rate (FDR) (< 1% FDR on protein and peptide spectrum match [PSM]). In addition to MaxQuant default settings, the search was performed against following variable modifications: Protein N‐terminal acetylation, pyroglutamate formation of glutamine, and oxidation (Met). Carbamidomethyl (Cys) was set as fixed modification. Arg6, Lys4, and Leu3 were set for medium SILAC labels and Arg10, Lys8, and Leu6 for heavy SILAC labels. Further data analysis was performed using R scripts developed in‐house. For quantification of pSILAC‐labeled proteins, the median was calculated from log_2_‐transformed normalized peptide heavy‐to‐medium ratios (H/M) for each protein. Two ratio counts were required for protein quantification. The mass spectra files and the corresponding data have been deposited at PRIDE (Accession number: PXD046891).

## 3 Statistical analysis

Data were analyzed using prism graphpad version 7 (GraphPad Software, Boston, MA, USA). Normal distribution of samples was tested with Shapiro–Wilk normality test. *P* values < 0.05 were considered significant (* for *P* < 0.05, ** for *P* < 0.01, *** for *P* < 0.001, **** for *P* < 0.0001).

## 4 Results

### 4.1 Surface HLA‐I expression is increased upon p14 knockdown

**Fig. 1:**
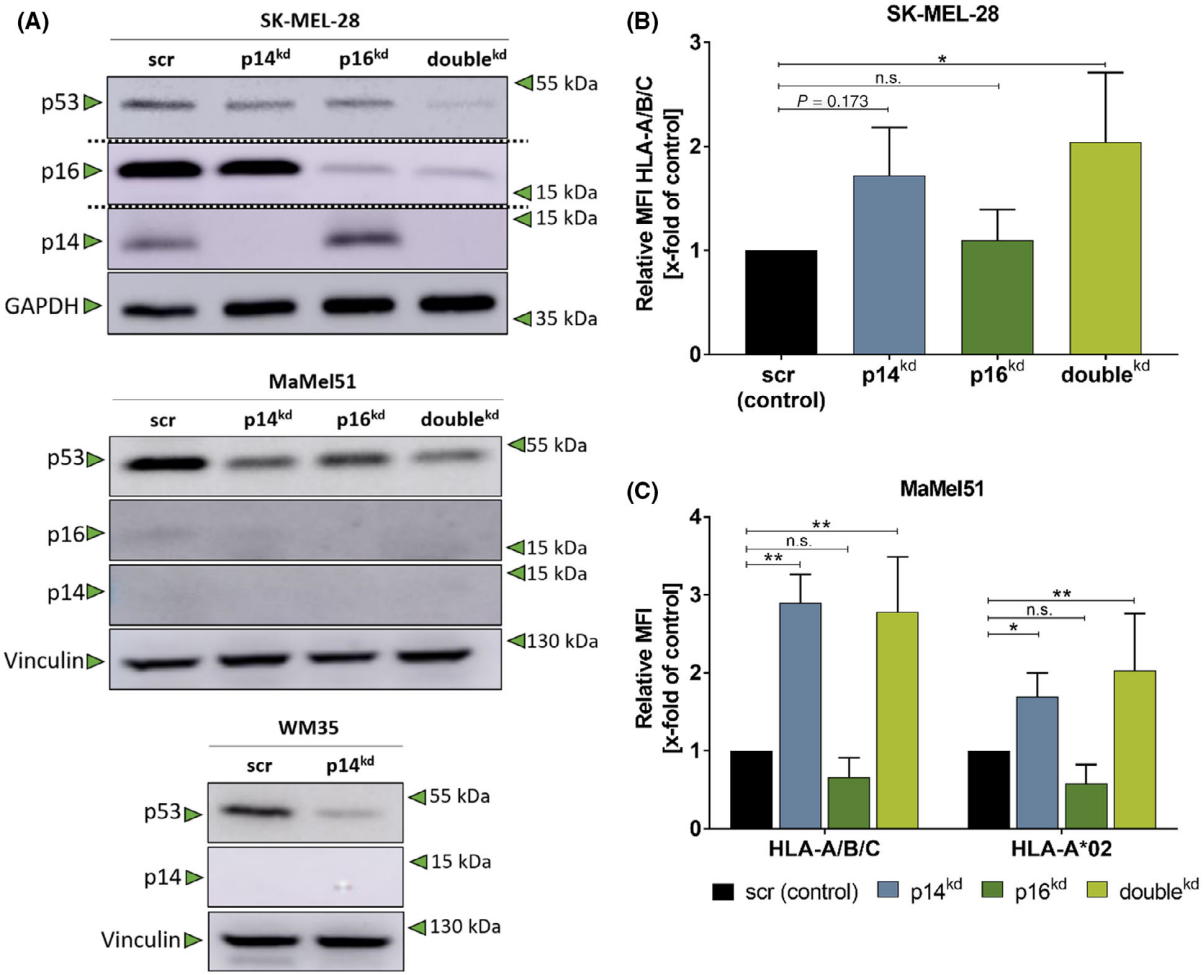
Knockdown of p14 is associated with increased HLA‐I expression. (A) short hairpin RNA (shRNA)‐mediated knockdown (kd) targeting p14 and/or p16 leads to significant downregulation of p14, p16 or both in melanoma cell lines. Western blot analysis of whole cell lysates of melanoma cell lines SK‐MEL‐28, MaMel51, and WM35 cells after shRNA‐mediated knockdown (6 days of doxycycline) of p14 (all), p16 or both (SK‐MEL‐28, MaMel51) show significant knockdown of *CDKN2A* gene products p14 and p16. If abundance of p14 and p16 were low (MaMel51 and WM35), p53 served as a surrogate marker for p14. Vinculin and GAPDH were used as loading controls. Dotted lines separate bands from different lanes of the same blot. Representative Western blot of *n* = 3. (B, C) HLA‐A/B/C and HLA‐A*02 surface expression increases due to p14^kd^ and double^kd^. (B) HLA‐A/B/C mean fluorescence intensity (MFI) of SK‐MEL‐28. (C) HLA‐A/B/C and HLA‐A*02 MFI of MaMel51. (B, C) MFIs were normalized to scr control cells and significances were determined by one‐way analysis of variances (ANOVA) with subsequent Dunnett's multiple comparison test. (B) *n* = 3, mean + SD, (C) *n* = 5 for scr and p14^kd^ HLA‐A*02, *n* = 3 for others, mean + SD. *P* values < 0.05 were considered significant (* for *P* < 0.05, ** for *P* < 0.01).

The loss or mutations of *CDKN2A* are frequent events in the development of melanoma [12]. This event is accompanied by impaired immunogenicity reflected by decreased cytotoxic T cell infiltration [20, 21, 22]. To investigate if the differential and concerted downregulation of the *CDKN2A* gene products p14 and p16 results in changes of melanoma immunogenicity, melanoma cell lines MaMel51, SK‐MEL‐28, and WM35 were transduced with shRNAs directed against the *CDKN2A* gene products p14, p16, and both to reflect the (partial) loss of *CDKN2A* or with a scr control vector. As shown in Fig. 1A, SK‐MEL‐28 cells showed significant knockdown of p14 (p14^kd^), p16 (p16^kd^), and both (double^kd^) on protein levels. MaMel51 and WM35 cells, however, had low basal protein expression of p14 and p16; therefore, p53 protein expression was used as a surrogate marker for profound p14^kd^ (Fig. 1A). RNAseq analyses demonstrated that *CDKN2A* levels in MaMel51 were downregulated upon knockdown (Fig. S1A).

HLA‐I is crucial for CD8^+^ T cell recognition of transformed cells. Figure 1B,C show that p14^kd^ and p16^kd^ differently alter the expression of HLA‐A/B/C of the HLA‐A*02‐negative SK‐MEL‐28 cells and of HLA‐A/B/C and HLA‐A*02 on MaMel51 cells. While p16^kd^ does not change the expression of HLA‐I molecules significantly, p14^kd^ and the double^kd^ led to a significant increase of HLA‐I molecules on the surface. Upregulation of HLA‐A/B/C and HLA‐A*02 due to p14^kd^ has also been observed in the cell line WM35 (Fig. S1B).

### 4.2 Knockdown of p14 in melanoma cells impairs T cell recognition

As these findings suggested a so far uninvestigated influence of p14 on melanoma immunogenic visibility, we addressed this topic in the following experiments.

Recognition and killing of transformed cells by cytotoxic (CD8^+^) T cells are crucial in effective tumor cell clearance. To exert effector functions, T cells have to recognize their cognate antigen presented on an HLA‐I molecule on the aberrant cells. Increased HLA‐I expression upon p14^kd^ (Fig. 1B,C, Fig. S1B) suggested a possibly increased immunogenicity. To investigate melanoma‐specific T cell interactions, HLA‐A*02:01‐specific T cell receptor‐transgenic T cells (TCR T cells) directed against the MDAs gp100_154‐162_ (T_gp100_) and MART‐1_27‐35_ (T_MART_) were generated. Lentiviral transduction of primary T cells resulted in high yields of antigen‐specific TCR T cells (Fig. S2A). High specificity of TCR T cells was assured in T2 peptide‐pulsing cocultures with T_gp100_ or T_MART_ as IFNg secretion was only observed in relevant peptide‐pulsed cocultures (Fig. S2B).

**Fig. 2:**
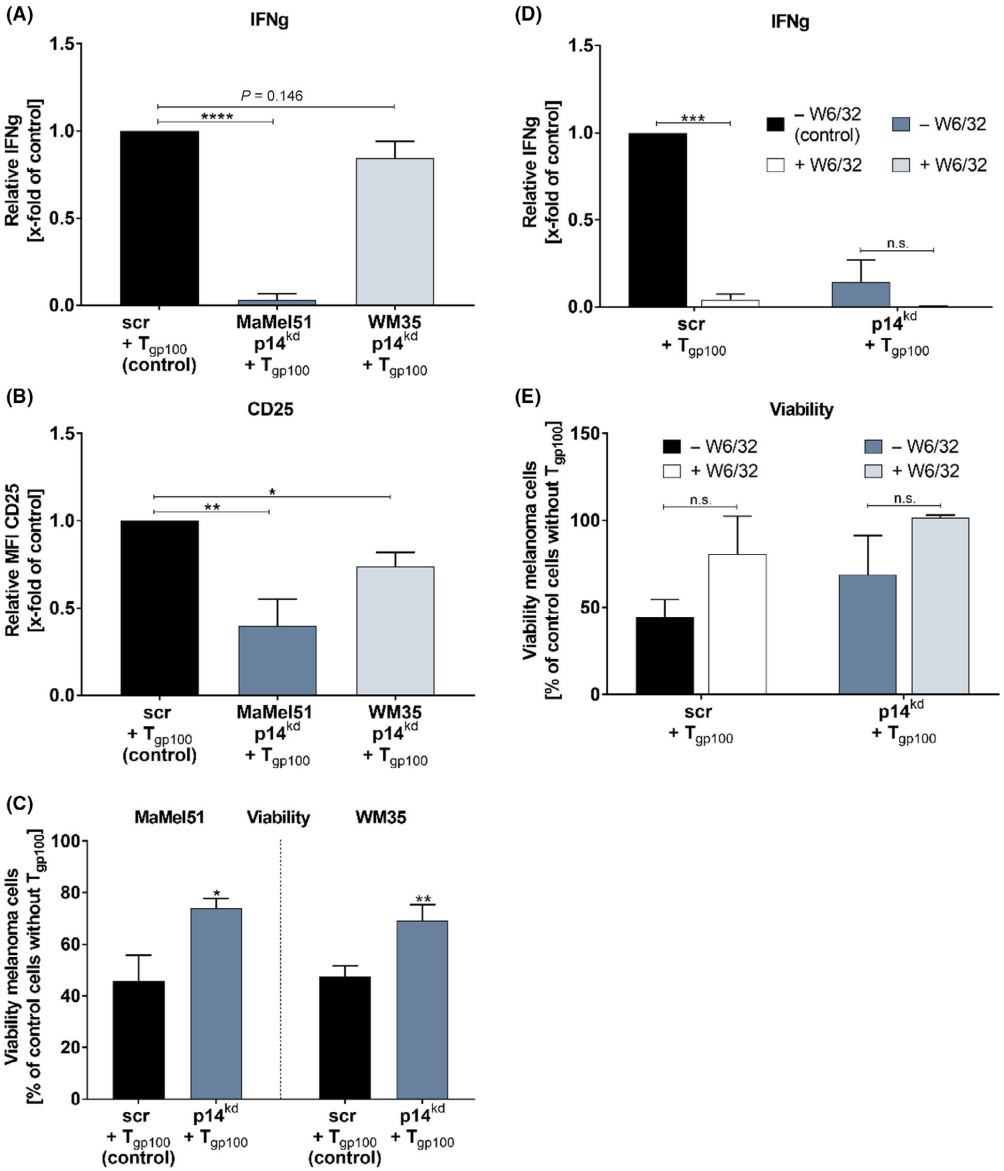
p14^kd^ of melanoma cells renders cells less sensitive to melanoma differentiation antigen‐specific T cell receptor‐transgenic T cells (TCR T cells). (A–C) p14^kd^ of MaMel51 and WM35 cells leads to impaired recognition, activation, and killing of T_gp100_ cells. (A) Measurement of Interferon gamma (IFNg) secretion, (B) mean fluorescence intensity (MFI) of CD25 T cell activation surface marker and (C) melanoma cell killing by Alamar Blue after coculture of MaMel51 and WM35 scr or p14^kd^ cells with T_gp100_ cells. (A) IFNg and (B) CD25 levels were normalized to scr control cells. (A–C) Significances were determined by unpaired, two‐tailed *t* test. (A, B) *n* = 2 for WM35, *n* = 3 for MaMel51, mean + SD (C) *n* = 3, mean + SD. (D, E) Blocking of HLA‐I in cocultures impairs interaction of T_gp100_ cells and MaMel51 scr and p14^kd^ cells. (D) Measurement of IFNg secretion and (E) melanoma cell killing by Alamar Blue after coculture with T_gp100_ cells + − HLA‐I blocking W6/32 antibody. (D) IFNg levels were normalized to scr control cells. (D, E) Significances were determined by one‐way analysis of variances (ANOVA) with subsequent Sidak's multiple comparison test. *n* = 2, mean + SD. *P* values < 0.05 were considered significant (* for *P* < 0.05, ** for *P* < 0.01, *** for *P* < 0.001, **** for *P* < 0.0001).

To determine immunogenicity, HLA‐A*02:01‐specific T_gp100_ cells were cocultured with the HLA‐A*02:01‐positive MaMel51 and WM35 cells harboring p14^kd^ or a scr control vector. Surprisingly, secretion of IFNg was significantly impaired upon coculture of T_gp100_ T cells with MaMel51 p14^kd^ cells while upon coculture with WM35 p14^kd^ cells a small but nonsignificant reduction compared to coculture with scr cells was observed (Fig. 2A). However, decreased CD25 expression on T cells implied dampened TCR T cell recognition and T cell activation (Fig. 2B). Consequently, melanoma cell viability of p14^kd^ cells was significantly higher than that of scr cells after cocultures (Fig. 2C). Importantly, restricting the HLA‐I axis in cocultures with MaMel51 cells by blocking antibodies (W6/32) diminished TCR T cell recognition and killing of scr and p14^kd^ cells, as measured by IFNg secretion and melanoma viability, thus implying an HLA‐I‐driven interaction (Fig. 2D,E).

Notably, recognition by T_MART_ cells upon coculture with melanoma cells was also impaired by p14^kd^ in melanoma cells albeit not as strong as recognition by T_gp100_ cells (Fig. S3).

In summary, despite increased HLA‐I expression upon p14^kd^ in melanoma cells, activation of T cells was found to be decreased and in one setting almost complete ignorance of melanoma target cells was observed.

### 4.3 p14 knockdown diminishes expression of melanoma differentiation antigens

**Fig. 3:**
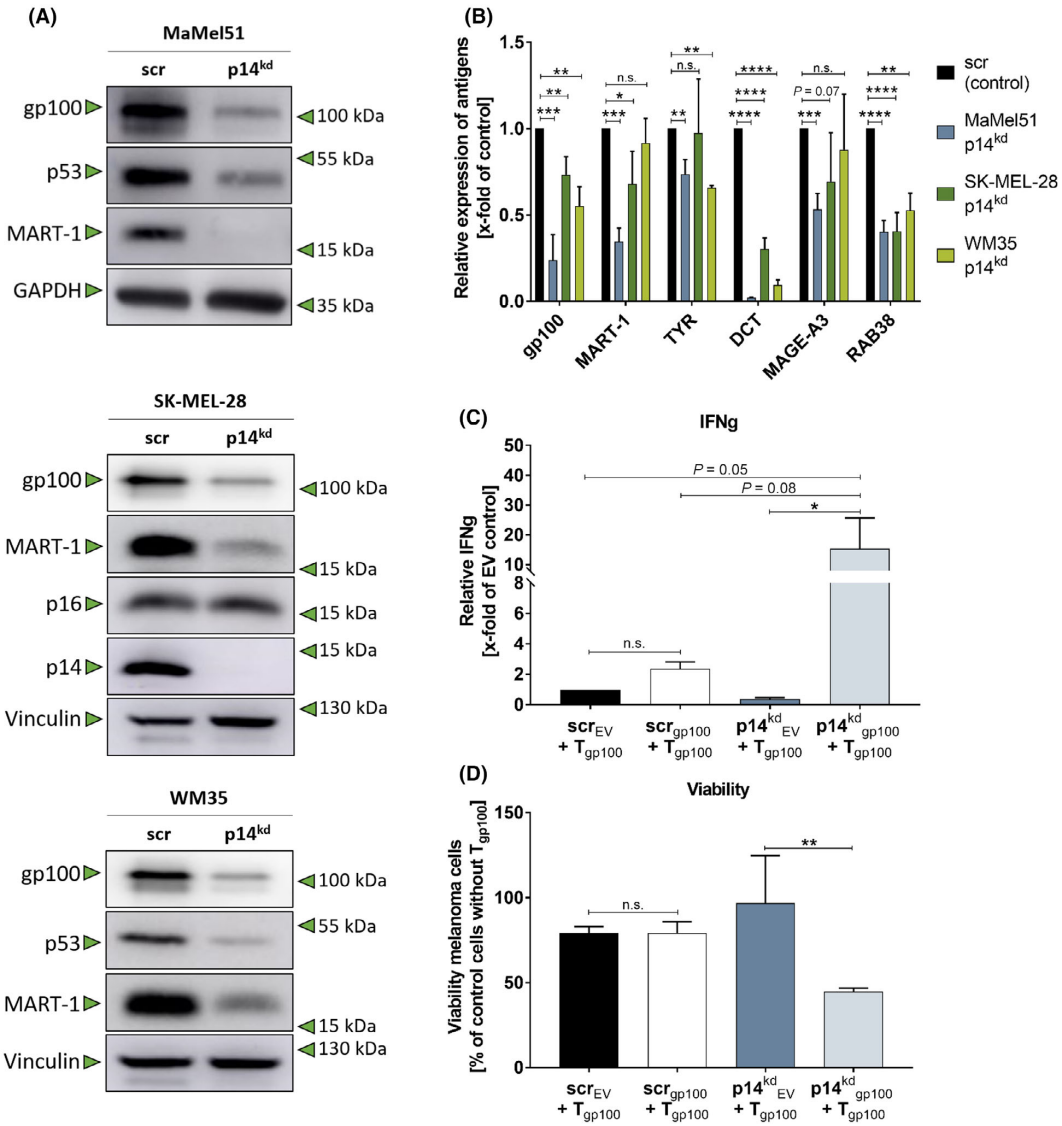
Melanoma (differentiation) antigens are downregulated upon p14^kd^. (A, B) Melanoma (differentiation) antigens are downregulated in melanoma cell lines due to p14^kd^. (A) Western blot analysis of whole cell lysates of melanoma cell lines MaMel51, SK‐MEL‐28 and WM35 with scr or p14^kd^. Vinculin and GAPDH were used as loading controls. Representative Western blot of *n* = 5 for SK‐MEL‐28 or *n* = 3 for others. (B) qPCR of MaMel51, SK‐MEL‐28 and WM35 cells with scr or p14^kd^. Gene expression of p14^kd^ cells was normalized to scr control cells of each respective cell line. *RPLP0* was used as a reference gene. Significances were determined by unpaired, two‐tailed t tests for scr and p14^kd^ cells of each respective cell line. *n* = 3, mean + SD. (C, D) Stable ectopic expression of gp100 enhances T_gp100_ cell recognition and killing of MaMel51 p14^kd^ compared to scr cells. (C) Measurement of Interferon gamma (IFNg) secretion, and (D) melanoma cell killing by Alamar Blue after coculture of MaMel51 scr or p14^kd^ cells with ectopic gp100 expression or an empty vector control (EV) with T_gp100_ cells. (C) IFNg values were normalized to scr_EV_ control cells. (C, D) Significances were determined by one‐way analysis of variances (ANOVA) and subsequent Sidak's multiple comparison test. *n* = 3, mean + SD. *P* values < 0.05 were considered significant (* for *P* < 0.05, ** for *P* < 0.01, *** for *P* < 0.001, **** for *P* < 0.0001).

Surprisingly, enhanced HLA‐I expression in p14^kd^ cells did not lead to improved TCR T cell recognitions but to nearly complete ignorance of melanoma target cells. Since HLA‐I expression levels were obviously not causal for reduced T cell recognition, the expression of the antigens gp100 and MART‐1 was investigated. Indeed, immunoblot analysis demonstrated that the MDAs gp100 and MART‐1 were downregulated upon p14^kd^ in the melanoma cell lines MaMel51, SK‐MEL‐28 and WM35 (Fig. 3A,B). A further real‐time PCR analysis of a wider panel of melanoma (differentiation) antigens confirmed that the melanoma differentiation antigen group and MAGE‐A3 were downregulated upon p14^kd^ (Fig. 3B). Moreover, reduced gp100 expression was also observed when targeting *CDKN2A* by siRNA in MaMel51 and SK‐MEL‐28 cells (Fig. S4A). In MaMel51, this was associated with a reduction in T_gp100_ cell recognition as indicated by impaired IFNg secretion (Fig. S4B).

In MaMel51 p14^kd^ cells, stable integration of a gp100 expression construct led to gp100 expression levels similar to parental cells (Fig. S4C). Furthermore, this ectopic gp100 expression could significantly increase recognition and killing of p14^kd^ MaMel51 melanoma cells while killing of scr cells in cocultures with T_gp100_ cells was unaffected (Fig. 3C,D). This implies that the surplus of HLA‐I molecules in p14^kd^ cells was loaded with gp100 peptides leading to a higher absolute and relative surface presentation of the peptides.

Besides the evident downregulation of MDA target antigens upon p14kd, upregulation of PD‐L1 was observed in p14kd cells causing additional dampening of the T cell‐mediated immune response toward target cells (Fig. S5).

### 4.4 Melanoma differentiation peptide density is decreased by p14 knockdown

**Fig. 4:**
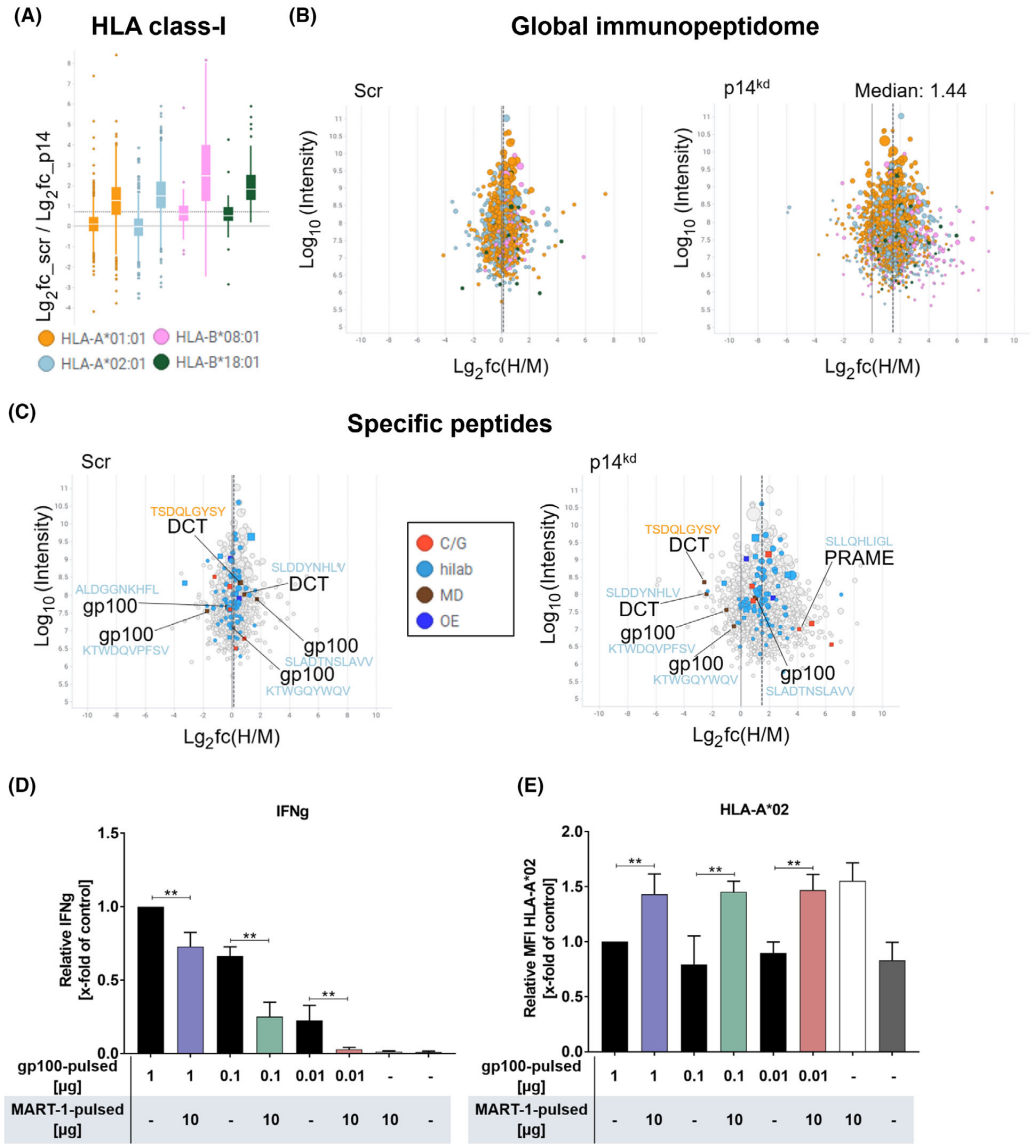
Melanoma differentiation antigen peptide density is affected by p14^kd^. (A–C) MDAs do not follow the trend of global upregulation of the immunopeptidome due to p14^kd^. (A) Expression of HLA‐A*01:01, HLA‐A*02:01, HLA‐B*08:01, and HLA‐B*18:01 and (B) the global immunopeptidome enhances upon p14^kd^ in comparison to scr control cells. (C) MDAs like gp100 and dopachrome tautomerase (DCT) demonstrate downregulation upon p14^kd^ compared to scr control cells. Immunopeptidomic analysis by liquid chromatography‐mass spectrometry (LC–MS) after 48 h of pulsed stable isotope labeling and amino acids in cell culture (pSILAC) treatment of MaMel51 scr and p14^kd^ cells. (A) Logarithmic fold change in abundancy of HLA‐I expression of scr control cells and p14^kd^ cells is shown. Median with 25^th^ and 75^th^ percentile is shown in box plots with whiskers representing minimum and maximum (1.5× interquartile range (IQR) from box end) and individual datapoints representing outliers (> 1.5× IQR from box end). (B) Logarithmic fold changes in heavy to medium‐labeled immunopeptides of scr control cells and p14^kd^ cells are shown. (C) Logarithmic fold changes in heavy to medium‐labeled specific immunopeptides of scr control cells and p14^kd^ cells are shown. C/G: Cancer/testis antigen; hilab: related to melanoma; MD: MDAs; OE: overexpressed in cancer. (D), (E) Antigen surface density affects T cell receptor‐transgenic T cell (TCR T cell) recognition. (D) Measurement of Interferon gamma (IFNg) secretion after peptide‐pulsing of MaMel51 with relevant (gp100_154‐162_) or irrelevant (MART‐1_27‐35_) peptide or a spiked combination of both in indicated concentrations and (D) coculture with T_gp100_ cells or (E) surface staining of HLA‐A*02 after peptide‐pulsing with gp100_154‐162_ or MART‐1_27‐35_ peptide or a spiked combination of both in indicated concentrations. Values were normalized to T2 gp100_154‐162_ pulsed [1 μg] control cells. Significances were determined by analysis of variances (ANOVA) with subsequent Sidak's multiple comparisons test. (D) *n* = 3 for T2 gp100 0.1 μg, T2 gp100/MART 0.1 μg/10 μg, T2 gp100 0.01 μg, T2 gp100/MART 0.01 μg/10 μg, *n* = 4 for others, mean + SD (E) *n* = 2 for T2 gp100 0.1 μg, T2 gp100/MART 0.1 μg/10 μg, T2 gp100 0.01 μg, T2 gp100/MART 0.01 μg/10 μg, *n* = 3 for T2 unpulsed, *n* = 4 for others, mean + SD. *P* values < 0.05 were considered significant (** for *P* < 0.01).

To elucidate whether the reduced gp100 expression upon p14^kd^ was part of a global reduction in proteins ending up in the MHC class‐I antigen presentation pathway, we next analyzed the global immunopeptidome by LC–MS after pSILAC treatment of MaMel51 scr and p14^kd^ cells. These analyses revealed that the upregulation of HLA‐I molecules (Fig. 4A) seen in p14^kd^ cells in comparison to scr control cells goes along with an increase of the global immunopeptidome (Fig. 4B). Alongside with the global upregulation of immune peptides, the CTA PRAME was more abundant in p14^kd^ cells (Fig. 4C). However, the MDAs gp100 and DCT did not follow the trend of upregulation leading to their less dense surface abundance. Accompanying to the shift in antigen presentation, the proteome analysis of MaMel51 p14^kd^ cells showed that expression of several other MDA proteins besides gp100 is downregulated upon p14^kd^. This confirms that not only the display but also the expression of peptide proteins was affected by the knockdown (Fig. S6).

To verify that surface density of presented peptides is playing a role in T cell recognition, T2 cells were peptide‐pulsed with different amounts of relevant (gp100_154‐162_) and irrelevant (MART‐1_27‐35_) peptides and were cocultured with T_gp100_ cells. Indeed, reflecting recognition of pulsed T2 cells, IFNg levels in supernatants of cocultures demonstrated that T2 cells pulsed with relevant peptide were recognized dose‐dependently and significantly better than T2 cells pulsed with same amounts of relevant peptides but spiked with large amounts of irrelevant peptides (Fig. 4D). In spiked conditions, HLA‐A*02 expression increased significantly compared to conditions pulsed with relevant peptide, leading to a surface dilution effect for HLA‐A*02:01 presented gp100_154‐162_ peptide (Fig. 4E). Supporting these findings, gp100_154‐162_ peptide was estimated *in silico* via NetMHCpan‐4.1, a trained artificial neural network, to bind stronger to HLA‐A*02:01 than irrelevant MART‐1_27‐35_ peptide leading to complete HLA‐A*02:01 binding of gp100_154‐162_ (Fig. S7).

### 4.5 Altered immunogenicity shapes the outcome of melanoma patients with homozygous loss of *CDKN2A*

**Fig. 5:**
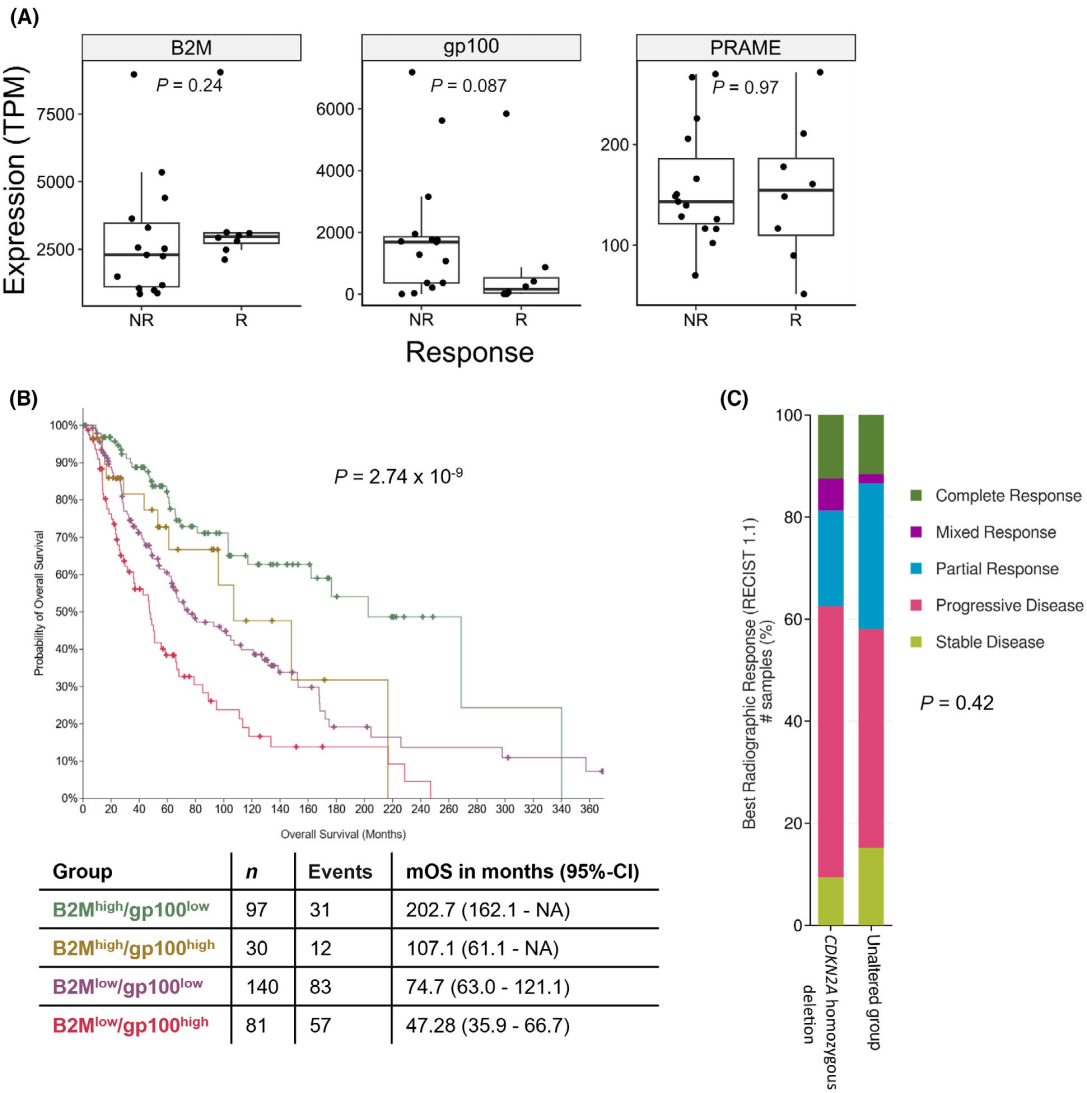
Immunogenic changes upon *CDKN2A* loss define the outcome of immune checkpoint inhibition. (A) Responder of immune checkpoint inhibition (ICI) with homozygous deletions in *CDKN2A* tend toward a Beta‐2 microglobulin (B2M) high, gp100 low phenotype. Bulk RNAseq analysis of 23 patients with homozygous deletions in *CDKN2A* stratified for response and nonresponse to PD‐1 immunotherapy. Transcript per million (TPM) values of *B2M*, *gp100* and *PRAME* were compared. Significances were determined by Wilcoxon test. NR, nonresponder; R, responder. (B) The overall survival (OS) of ICI patients is markedly increased upon the abundance of B2M and the absence of gp100. Survival analysis from 363 samples of cutaneous melanoma patients from cBioPortal [38]. Patients were stratified based on B2M and PMEL (gp100) expression and median OS (mOS) was calculated by the Kaplan–Meier Method. Significance was determined by log‐rank test. (C) Homozygous loss of *CDKN2A* does not alter the best radiographic response of ICI‐treated patients. Best radiographic response was categorized according to RECIST 1.1 in 144 melanoma patients who received PD‐1 blockade for advanced melanoma. Distribution of response categories (complete response, partial response, mixed response, stable disease, and progressive disease) was compared between patients with (*n* = 32) or without (*n* = 112) homozygous deletion of *CDKN2A* using the chi‐squared test.

To investigate the clinical implications of our *in vitro* data, pretreatment samples of 23 melanoma patients with homozygous loss of *CDKN2A* who received anti‐PD‐1 monotherapy were stratified by response and nonresponse and analyzed for transcriptional changes in the expression of B2M, the MDA gp100, and the CTA PRAME (Fig. 5A). The expression of the surrogate marker for antigen presentation B2M was nonsignificantly upregulated in responders compared to nonresponders. In contrast to this, the MDA gp100 demonstrated nonsignificant downregulation within responders compared to nonresponders, thus reflecting the observed phenotype of p14^kd^ melanoma cells. However, no significant difference could be observed in the expression of the CTA PRAME between responders and non‐responders.

Setting these data into a general perspective, overall survival (OS) data of melanoma patients demonstrated a significant survival benefit of patients with a B2M high and gp100 low phenotype (Fig. 5B). Markedly, the abundance of B2M had a strong positive impact on the OS of patients in general. Intriguingly, the presence of gp100 was inversely correlated with OS in ICI‐treated melanoma patients.

However, the homozygous loss of *CDKN2A* had no impact on best radiographic response of ICI patients compared to *CDKN2A* proficient patients (Fig. 5C).

In summary, these clinical data suggest that responding melanoma patients with a homozygous *CDKN2A* deletion show a trend toward the observed phenotype of increased antigen display and diversity despite a downregulation of the MDA gp100. Moreover, the OS of melanoma patients is significantly influenced by the absence of gp100 and especially the abundance of B2M.

### 4.6 Alterations of the immunogenic phenotype by p14^kd^ are p53‐dependent

**Fig. 6:**
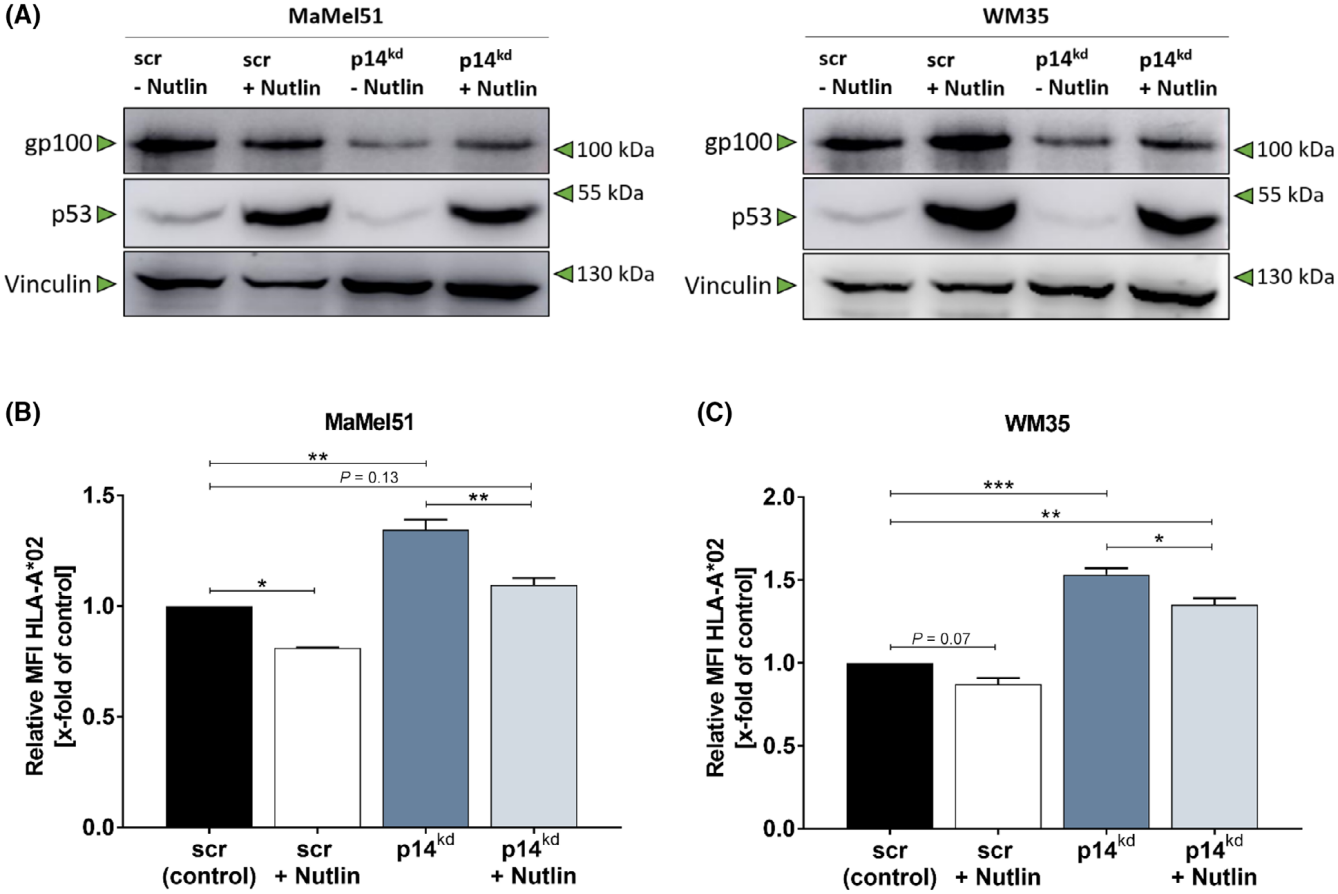
p14^kd^‐induced immunogenic changes are p53‐dependent. (A–C) Downregulation of gp100 and upregulation of HLA‐A*02 is p53‐dependent. (A) Western blot analysis of whole cell lysates of melanoma cell lines MaMel51 and WM35 with scr or p14^kd^ with or without Nutlin‐3 treatment. Vinculin was used as loading controls. Representative Western blot of *n* = 2. (B, C) HLA‐A*02 mean fluorescence intensity (MFI) of (B) MaMel51 and (C) WM35 cells with scr or p14^kd^ with or without Nutlin‐3 treatment. MFI was normalized to scr control cells and significance was determined by one‐way analysis of variances (ANOVA) with subsequent Sidak's multiple comparison test. *n* = 2, mean + SD. *P* values < 0.05 were considered significant (* for *P* < 0.05, ** for *P* < 0.01, *** for *P* < 0.001).

In a cellular stress scenario, the upregulation of p14 is accompanied with an accumulation of p53 and a subsequent cell cycle arrest or apoptosis [17]. The strong embedding of p53 in the signaling cascade of p14 and its emerging role in immune regulation suggested a p53‐driven mechanism taking place in p14^kd^ melanoma cells [45]. Therefore, the p14^kd^‐driven degradation of p53 was decreased by inhibition of the E3‐ubiquitine ligase MDM2 via Nutlin‐3 and gp100 protein as well as HLA‐A*02 expression levels were quantified in MaMel51 and WM35 cells. The blockade of MDM2‐induced p53 degradation led to an accumulation of p53 in the melanoma cells, reinduced the gp100 expression and downregulated HLA‐A*02 expression in p14^kd^ cells proving a p53‐dependent mode of action (Fig. 6A–C).

### 4.7 p14 knockdown induces non‐canonical Wnt signaling

**Fig. 7:**
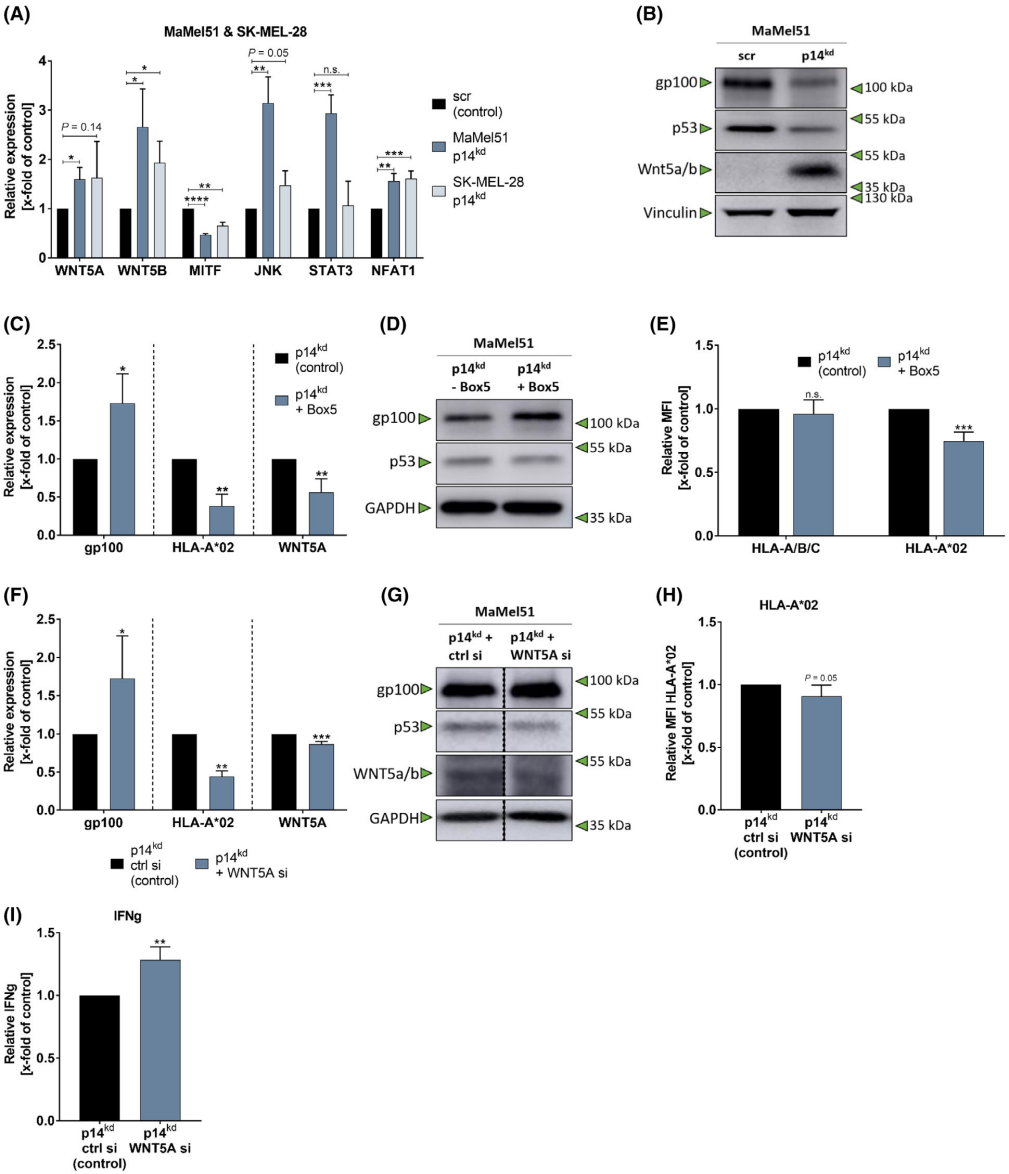
p14^kd^ induces non‐canonical Wnt5a signaling. (A, B) Knockdown of p14 leads to non‐canonical Wnt signaling. (A) qPCR of Wnt signaling‐related genes of MaMel51 and SK‐MEL‐28 scr and p14^kd^ melanoma cell lines. *RPLP0* was used as a reference gene. Fold change induction was normalized to scr control cells of each cell line and significances were determined by unpaired, two‐tailed t tests for scr and p14^kd^ cells of each cell line. *n* = 3 for SK‐MEL‐28 WNT5B, MITF, JNK, *n* = 4 for others, mean + SD. (B) Western blot analysis of whole cell lysates of MaMel51 scr and p14^kd^ melanoma cell line. Vinculin was used as a loading control. Representative Western blot of *n* = 3. (C–E) WNT5A signaling inhibition reverts p14^kd^‐mediated gp100 and HLA‐A*02 changes. (C) qPCR of MaMel51 p14^kd^ cells + − Box5 treatment. *RPLP0* was used as a reference gene. Fold change induction was normalized to p14^kd^ cells – Box5 and significances were determined by unpaired, two‐tailed t tests. *n* = 3, mean + SD. (D) Western blot analysis of whole cell lysates of MaMel51 p14^kd^ melanoma cell lines + − Box5. GAPDH was used as a loading control. Representative Western blot of *n* = 3. (E) Only HLA‐A*02 surface expression decreased due to Box5 treatment. HLA‐I surface expression mean fluorescences intensity (MFI) of p14^kd^ cells + − Box5 treatment. MFI was normalized to p14^kd^ cells – Box5 treatment and significances were determined by unpaired, two‐tailed t tests. *n* = 3, mean + SD. (F–H) *WNT5A* short interfering RNA (siRNA) counteracts the phenotype of p14^kd^. (F) qPCR of MaMel51 p14^kd^ cells with *WNT5A* siRNA or control siRNA. *RPLP0* was used as a reference gene. Fold change induction was normalized to p14^kd^ cells without *WNT5A* siRNA and significances were determined by unpaired, two‐tailed t tests. *n* = 2 for HLA‐A*02, *n* = 3 for gp100, *n* = 4 for WNT5A, mean + SD. (G) Western blot analysis of whole cell lysates of MaMel51 p14^kd^ melanoma cells with *WNT5A* siRNA or control siRNA. GAPDH was used as a loading control. Representative Western blot of *n* = 2. (H) HLA‐A*02 surface expression decreased due to *WNT5A* siRNA treatment. HLA‐A*02 surface expression MFI of p14^kd^ cells with *WNT5A* siRNA or control siRNA. MFI was normalized to p14^kd^ cells with control siRNA and significance was determined by unpaired, two‐tailed t test. *n* = 3, mean + SD. (I) *WNT5A* siRNA treatment of p14^kd^ melanoma cells improves recognition of T_gp100_ cells. Measurement of Interferon gamma (IFNg) secretion after coculture of MaMel51 p14^kd^ cells with *WNT5A* siRNA or ctrl siRNA and T_gp100_ cells. IFNg levels were normalized to p14^kd^ cells and significance was determined by unpaired, two‐tailed t test. *n* = 3, mean + SD. *P* values < 0.05 were considered significant (* for *P* < 0.05, ** for *P* < 0.01, *** for *P* < 0.001, **** for *P* < 0.0001).

Wnt5a signaling is known to reduce the expression of MDAs [46]. WNT5A can signal via different pathways. Among others, *STAT3*, *JNK*, and *NFATC2* (NFAT1) are downstream targets of WNT5A [46, 47]. Ultimately, downregulation of *MITF* due to Wnt5a signaling leads to the downregulation of MDAs [46]. Interestingly, qPCR demonstrated upregulation of many transcriptional targets of non‐canonical Wnt signaling in p14^kd^ cells (Fig. 7A). Moreover, immunoblot analysis revealed that WNT5A is strongly upregulated in MaMel51 cells upon expression of the p14‐targeting shRNA (Fig. 7B).

Given the upregulation of WNT5A signaling in p14^kd^ cells, investigations of WNT5A reducing agents on the phenotype of p14^kd^ cells were conducted with the final aim to enhance MDA‐specific T cell recognition of these cells.

Effective blockade of WNT5A in the HLA‐A*02:01‐positive cell line MaMel51 with p14^kd^ with the competitive WNT5A mimic Box5 reverted the phenotype of differentiation antigen downregulation (Fig. 7C,D) and HLA‐A*02 upregulation (Fig. 7E). By interruption of the positive Wnt5a feedback loop by inhibiting FZD5 via Box5, *WNT5A* transcription was downregulated (Fig. 7C) [48]. Interestingly, solely HLA‐A*02, not pan HLA‐I of p14^kd^ cells were affected by upregulation due to WNT5A inhibition (Fig. 7E). Due to short‐lived effects of Box5 treatment in cocultures of T_gp100_ and MaMel51 cells (Fig. S8), *WNT5A* siRNA was used to transiently knockdown *WNT5A* over the course of coculture in MaMel51 cells. *WNT5A* siRNA knockdown resulted in a downregulation of *WNT5A* expression (Fig. 7F,G). Further, gp100 as well as HLA‐A*02 levels reverted comparably to observations after Wnt5a inhibition (Fig. 7F–H). Accordingly, cocultures of T_gp100_ cells with p14^kd^/Wnt5a^kd^ MaMel51 cells showed a slight increase in T cell recognition, indicating a revertible phenotype (Fig. 7I).

In summary, these data demonstrate an association between the reduction of p14 and the downregulation of MDAs in melanoma cells via Wnt5a signaling. This has consequences for melanoma cell immunogenicity and might pose a role in ICI treatment of melanomas by rendering these cells less sensitive to MDA‐specific T cell engagements.

## 5 Discussion

The loss of *CDKN2A* is affecting the immune landscape of melanomas. In this study, we show that the repression of the *CDKN2A* gene product p14 results in dramatic alterations of the immunogenicity of melanoma cells via p53‐dependent signaling. Increasing the abundance of HLA‐I molecules and global immune peptides on the surface of p14 downregulated cells might represent an attempt of the cell to provoke better recognition by the immune system. Profound HLA‐I expression is a key factor for the prevention of cancerogenesis as it regulates invasion, migration, and grants immunogenicity to melanoma cells [49, 50]. In responding patients with a homozygous *CDKN2A* loss, we demonstrated an increase in the expression of the HLA class‐I surrogate marker B2M. This hints toward an enhanced display of a diverse set of antigens as an immunogenically diverse tumor correlates with better response to ICI [51]. This nonsignificant trend would most likely become more pronounced in a larger patient cohort. In line with that, increased HLA class‐I peptide presentation upon p14 loss could possibly be harnessed therapeutically. The antigen PRAME exhibited enhanced display in immunopeptidomic experiments of melanoma cell lines which predestines the CTA as a target for directed immunotherapeutic approaches. Further, the boosted HLA class‐I expression in p14 impaired tumors could be exploited by synergistically provoking the generation of neoantigens via combined chemotherapy/radiotherapy or oncolytic therapy with subsequent immunotherapy [52, 53, 54]. Successful enhancement of antigen presentation by this strategy was shown with the chemotherapeutic agent temozolomide in a glioblastoma model [55].

Intriguingly, class‐I upregulation is only one consequence of p14 repression. MDAs were found to be downregulated by p14^kd^ *in vitro*. Given the general upregulation of class‐I molecules in the p14^kd^ cells, MDA‐derived peptides become the needle in the haystack for MDA‐specific cytotoxic T cells. In homozygous *CDKN2A* loss patients, we demonstrated that the same phenotype, namely a decrease in the expression of gp100 with a concomitant upregulation of B2M, is beneficial for the response of ICI patients. Survival data of melanoma patients confirmed that this particular phenotype is of benefit for the patients. Further, the proteomic and immunopeptidomic data from p14^kd^ cell lines showed that also the global immunopeptidome increased, suggesting a more diverse peptide surface pool. This implies that in an ICI treatment scenario, where a diverse T cell repertoire is beneficial for outcome, the relevance of the individual peptide might play a subordinate role. Interestingly, the expression of gp100 is an inverse determinant for the OS of ICI patients. Kim et al. [56] found that this phenotype might be a result of continuous IFNg exposure. However, we observed the decline of MDAs in *in vitro* experiments without the influence of IFNg, thus implying additional factors in generating this phenotype. The presence of gp100 on melanoma cells and therefore their differentiation status might represent a state of tolerance conserved from differentiated melanocytes [57].

However, studies have found a correlation between a high density of presented surface peptides and the activation of cognate cytotoxic T cells [58, 59, 60]. In p14^kd^ melanoma cells, the differentiation peptide surface density is low, rendering the cells nearly invisible for cognate cytotoxic T cells. This implies possible consequences in MDA‐directed therapeutic settings for melanoma patient subgroups characterized by p14 loss of function. The underrepresentation of MDA might render these patients less responsive to vaccination or adoptive T cell transfer strategies that target these antigens. Additionally, elevated PD‐L1 surface levels were found upon p14^kd^ in melanoma cell lines, which might result in additional dampening of the T cell response. In the last decade, TCR‐engineered T cells and cancer vaccines against differentiation antigens showed disappointing response rates in melanoma which might be partially due to observed genetic reasons [61, 62, 63]. With the advent of mRNA vaccines, however, personalized approaches for vaccine development could circumvent low abundant surface peptide presentations [64]. The results of the KEYNOTE‐942 trial further underline the significance of inducing a patient‐specific neoantigenic immune response [65]. The phase 2 trial proved a superior recurrence free survival and distant metastastis free survival for the combination of a novel mRNA vaccine and pembrolizumab versus pembrolizumab alone in high‐risk melanoma patients.

ICIs are the gold standard in modern melanoma treatment due to durable responses and survival benefits [2, 3]. Nevertheless, ICI efficacy eventually relies on stable antigen engagement by reactivated cytotoxic T cells [66, 67]. Absence or low abundance of cognate presented antigens, however, could dampen ICI response rates in melanoma patients. Clinical assessments of outcome parameters of patients with *CDKN2A* alterations are divergent as some studies showed worse outcome or no correlation of ICI outcome with *CDKN2A* alterations [18, 68]. In hereditary melanoma, however, *CDKN2A* alterations seem to be beneficial for response to ICI hinting toward a larger neoantigen repertoire in the tumors of this small patient group underlining the importance of proper antigen presentation [69].

In most of these cases, the whole *CDKN2A* gene locus or both gene products were considered as alterations by these studies, as these are the most common events found in melanoma according to TCGA analyses and studies [12, 70, 71]. In our analyzed cohort, the absence of the *CDKN2A* locus could not be identified as a biomarker for best radiographic response of ICI patients. This demonstrates that a more sophisticated look at the *CDKN2A* gene products is warranted. The real‐life implications of the rarer event of sole p14 inactivation in melanoma are still unclear [12]. Our data encourage the closer, more differentiated look at *CDKN2A* alterations as the different gene products have divergent implications on immunogenicity.

It is also to be considered that the loss of whole bands of chromosome 9 is accompanied by a loss of JAK2 which plays an important role in mediating IFNg signaling, and the adjacent important IFN type‐I gene cluster. The loss of these gene clusters is correlated with worse outcome in melanoma [72, 73, 74]. Our data show that the sole downregulation of p14, regardless of JAK signaling impairments, already has a major impact on the immunogenicity of melanoma cells.

Resulting in the reduction of MDAs, the process of dedifferentiation marks a common feature of progressing melanomas. Dissanayake et al. [46] showed that downregulation of differentiation antigens is driven by WNT5A‐mediated *MITF* reduction and affects differentiation antigen‐specific T cell interaction. For the first time, we show a direct link between impaired p14 expression and the described downregulation of MDAs via Wnt5a signaling. Reducing p14 in melanoma cell lines induced non‐canonical Wnt signaling via WNT5A and ultimately led to downregulation of differentiation antigens. Both the pharmacological WNT5A mimic Box5 and *WNT5A* siRNA could revert this p14^kd^ driven phenotype. However, Wnt5a blockade in a therapeutical setting might be a two‐sided medal. As melanoma cells re‐express differentiation antigens due to Wnt5a blockade, HLA‐A*02 molecules were found to be downregulated by this as well. Despite having differentiation antigens present on the surface, this might result in a less diverse cytotoxic T cell population which is correlated with a worse patient outcome [51, 75, 76]. Sequential treatment of p14 loss of function patients with non‐canonical Wnt signaling inhibitors, however, could create a broader T cell repertoire by bringing back underrepresented differentiation antigens to trigger cognate T cells. A few non‐canonical Wnt signaling intercalators are in early clinical trials for melanoma to address the relevance of Wnt signaling in a therapeutical context [77, 78].

## 6 Conclusions

In this study, we show that specific knockdown of the *CDKN2A* gene product p14 leads to the downregulation of MDAs and to an overall increase of HLA‐I surface molecules in melanoma cell lines. With this significant shift in peptide surface density, MDA‐specific T cells are heavily impaired in recognizing and killing affected melanoma cells. However, ICI patients with the respective phenotype demonstrate improved response and survival suggesting a more diverse immunopeptidome. Inhibition of the activated WNT5A signaling pathway in p14 knockdown melanoma cells reverts this phenotype. Our results show a new link between the alteration of p14 and the immunogenicity of melanoma cells. This has implications for melanoma patients with the respective genetic background.

## Conflict of interest

DL reports receiving honorarium and travel expenses from Genentech and is on the scientific advisory board of Oncovalent Therapeutics, neither of which have relevant interests associated with the results nor conduct of this study.

## Author contributions

JW conceived, designed, supervised the study, acquired data, conducted data analysis and interpretation, and wrote the manuscript. CK conceived, designed, and supervised the study, interpreted the data, and provided material. DF, MBert, CS, and MBern acquired data and were involved in methodology development and data analysis. AS, DSchr, RH, and SM participated in methodology development and contributed to study conception and design. TA and DL conducted data analysis. DScha provided material and contributed to study conception and design. MG provided material and facilities and contributed to study conception and design. BS conceived, designed, and supervised the study, interpreted the data, developed methodology, provided material, funding, and facilities, and participated in writing the manuscript. All authors contributed to the review of the manuscript.

### Peer review

The peer review history for this article is available at https://www.webofscience.com/api/gateway/wos/peer‐review/10.1002/1878‐0261.13660.

## Supporting information


**Fig. S1.** Knockdown of p14 is efficient and triggers HLA‐I upregulation in melanoma cell lines.
**Fig. S2.** T cell receptor‐transgenic T cells (TCR T cells) are a tool to measure immunogenicity of target cells.
**Fig. S3.** p14^kd^ melanoma cells are less recognized by MART‐1 specific TCR T cells.
**Fig. S4.** Melanoma differentiation antigens are affected by knockdown of *CDKN2A* and its gene products.
**Fig. S5.** Knockdown of p14 upregulates PD‐L1 expression.
**Fig. S6.** Melanoma differentiation antigen (MDA) protein expression is altered upon p14^kd^.
**Fig. S7.** *In silico* peptide‐binding capacity might define binding probability.
**Fig. S8.** T_gp100_ cell recognition of p14^kd^ cells is not affected by Box5 treatment.

**Table S1.** Cell line characteristics.

**Table S2.** Oligonucleotide sequences.

**Table S3.** Peptide sequences.

## Data Availability

The data that support the findings of this study are available in the Supporting Information material of this article. RNAseq data of melanoma cell lines are deposited at GEO (Accession number: GSE249763). The mass spectra files of pSILAC experiments and the corresponding data have been deposited at PRIDE (Accession number: PXD046891).
