# Supplementary material for: Loss of p14 diminishes immunogenicity in melanoma via non‐canonical Wnt signaling by reducing the peptide surface density
Source: Mol Oncol. 2024 May 28;18(10):2449–70. doi: 10.1002/1878-0261.13660 (PMC11459041; doi:10.1002/1878-0261.13660)
Supplement: Supplementary file 1 — Fig. S1. Knockdown of p14 is efficient and triggers HLA‐I upregulation in melanoma cell lines. Fig. S2. T cell receptor‐transgenic T cells (TCR T cells) are a tool to measure immunogenicity of target cells. Fig. S3. p14kd melanoma cells are less recognized by MART‐1 specific TCR T cells. Fig. S4. Melanoma differentiation antigens are affected by knockdown of CDKN2A and its gene products. Fig. S5. Knockdown of p14 upregulates PD‐L1 expression. Fig. S6. Melanoma differentiation antigen (MDA) protein expression is altered upon p14kd. Fig. S7. In silico peptide‐binding capacity might define binding probability. Fig. S8. Tgp100 cell recognition of p14kd cells is not affected by Box5 treatment. [file MOL2-18-2449-s004.docx]

# Supplementary Figures

## Figure S1
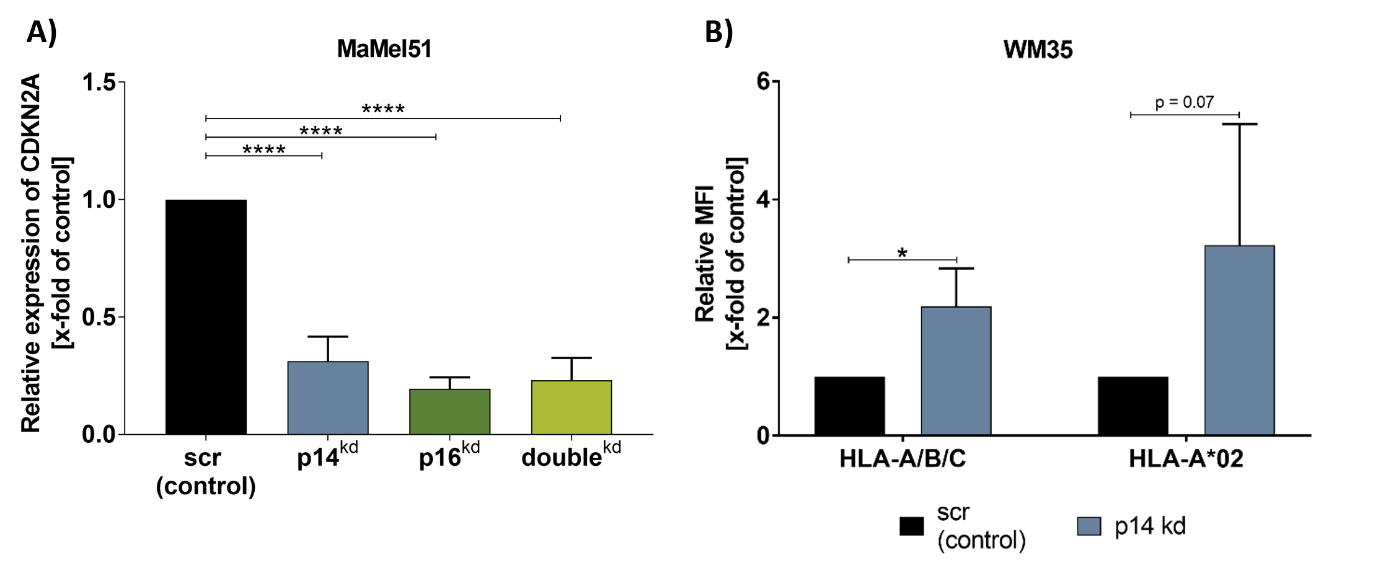


**Figure S1: Knockdown of p14 is efficient and triggers HLA-I upregulation in melanoma cell lines.**

**(A)** Knockdown of p14, p16 and both significantly reduces CDKN2A expression. RNA sequencing (RNAseq) analysis of CDKN2A knockdown in MaMel51 p14^kd^, p16^kd^ and double^kd^ knockdown cell lines. Relative expression was normalized to scr control cells and significance was determined by one-way analysis of variance (ANOVA) with subsequent Dunnett‘s multiple comparison test. n=3, mean + standard deviation (SD).
**(B)** HLA-I surface expression is upregulated due to p14^kd^ in WM35 melanoma cell line. HLA-A/B/C and HLA-A*02 mean fluorescence intensity (MFI) of WM35 scr and p14^kd^ cells. MFI was normalized to scr control cells and significances were determined by unpaired, two-tailed t tests. n=4, mean + SD. p values < 0.05 were considered significant (* for p < 0.05, **** for p < 0.0001).

## Figure S2


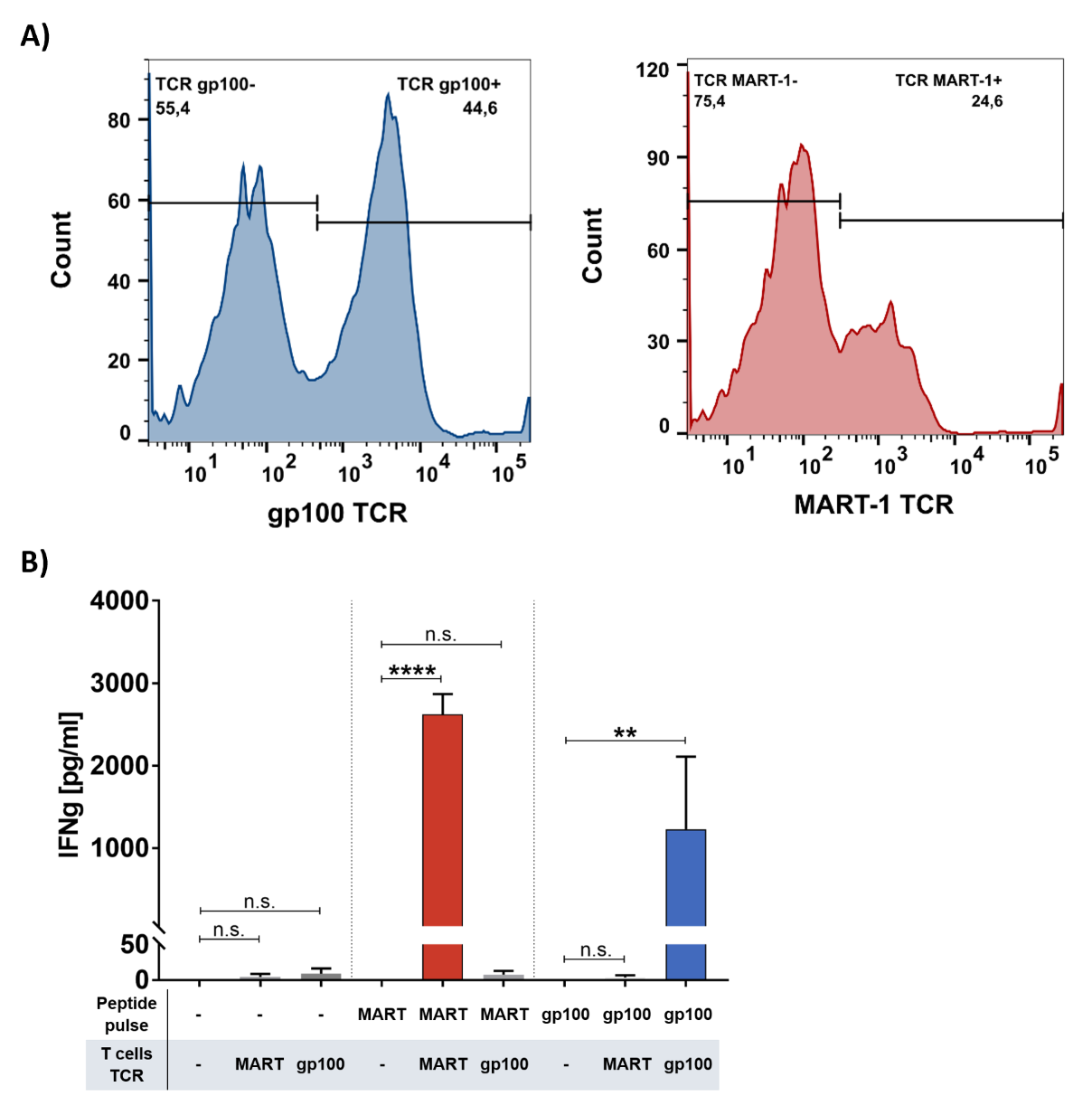


**Figure S2: T cell receptor-transgenic T cells (TCR T cells) are a tool to measure immunogenicity of target cells.**

**(A)**, **(B)** High yields of highly antigen-specific TCR T cells were generated. **(A)** Mean fluorescence intensity (MFI) of surface staining of T_gp100_ and T_MART_ cells with gp100- and MART-1-specific major histocompatibility complex (MHC) tetramer. Representative flow cytometry blots of tetramer stainings for gp100 and MART-1 specific T cell receptors (TCRs). **(B)** T_gp100_ and T_MART_ showed highly specific recognition of target antigen-pulsed T2 cells. Measurement of Interferon gamma (IFNg) levels of supernatants after cocultures of gp100_154-162_ or MART-1_27-35_ peptide pulsed T2 cells with T_gp100_ or T_MART_ cells. Significances were determined by one-way analysis of variance (ANOVA) and subsequent Sidak’s multiple comparison test. n=2 for T2 unpulsed, T2 MART, T2 MART + T_MART_, T2 gp100, n=3 for others, mean + SD. p values < 0.05 were considered significant (** for p < 0.01, **** for p < 0.0001).

## Figure S3


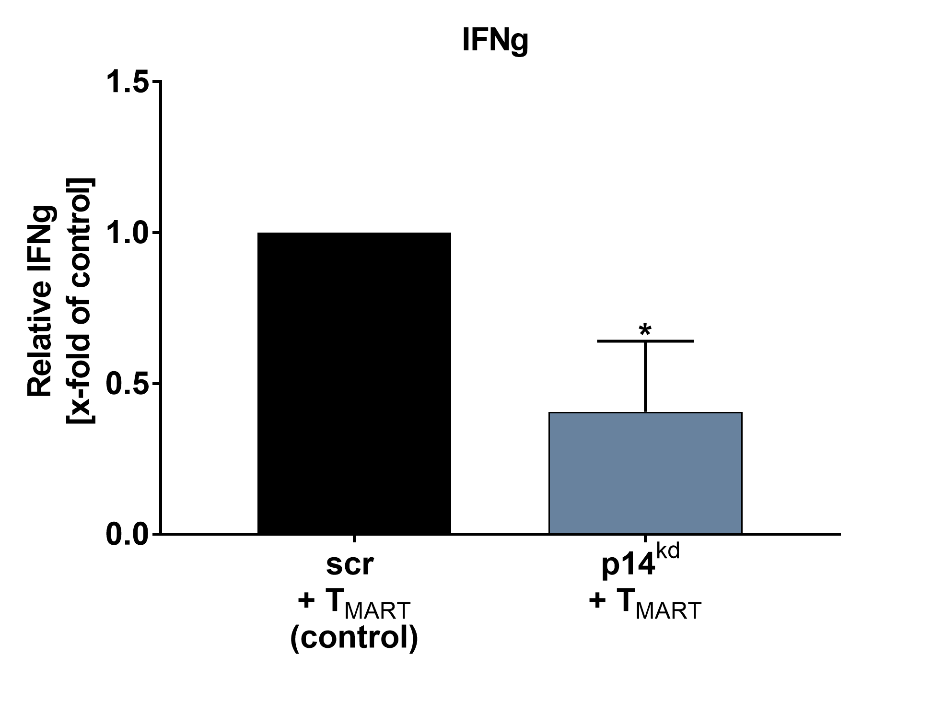


**Figure S3: p14^kd^ melanoma cells are less recognized by MART-1 specific TCR T cells.**

Knockdown of p14 also impairs recognition by T_MART_. Measurement of Interferon gamma (IFNg) secretion after coculture of MaMel51 with T_MART_. IFNg levels were normalized to scr control cells and significance was determined by unpaired, two-tailed t test. n=3, mean + SD. p values < 0.05 were considered significant (* for p < 0.05).

## Figure S4
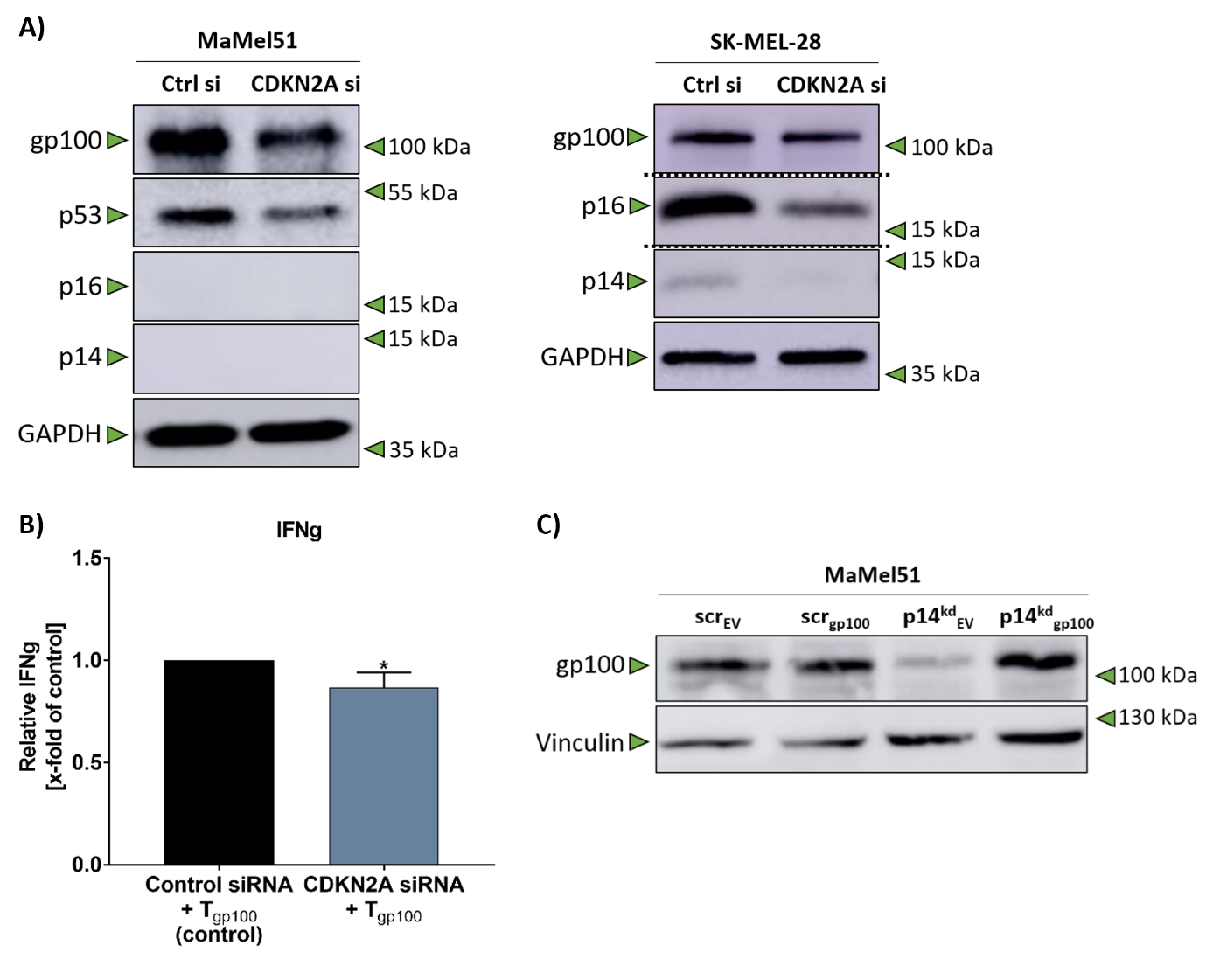


**Figure S4:** **Melanoma differentiation antigens are affected by knockdown of CDKN2A and its gene products.**

**(A)**, **(B)** short interfering RNA (siRNA)-mediated knockdown of CDKN2A results in downregulation of gp100 and impaired T cell receptor-transgenic T cell (TCR T cell) recognition of melanoma cells. **(A)** Western blot analysis of whole cell lysates of MaMel51 and SK-MEL-28 melanoma cell lines after CDKN2A siRNA or control siRNA treatment. GAPDH was used as loading control. Representative Western blot of n=3. **(B)** Measurement of Interferon gamma (IFNg) secretion after coculture of MaMel51 cells with CDKN2A siRNA or control siRNA and T_gp100_. IFNg levels were normalized to control siRNA cells and significance was determined by unpaired, two-tailed t test. n=3, mean + SD. **(C)** Overexpression of gp100 rescues gp100 protein expression in MaMel51 p14^kd^. Western blot analysis of whole cell lysates of MaMel51 scr or p14^kd^ cells with empty vector (EV) or gp100 overexpression vector (gp100). Vinculin was used as loading control. Representative Western blot of n=3. p values < 0.05 were considered significant (* for p < 0.05).

## Figure S5


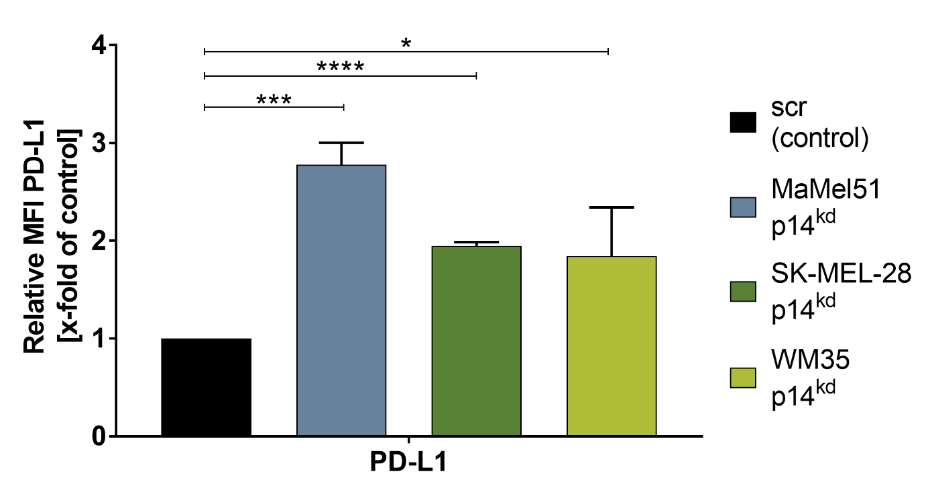


**Figure S5:** **Knockdown of p14 upregulates PD-L1 expression.**

PD-L1 surface expression is upregulated due to p14^kd^ in several melanoma cell lines. PD-L1 mean fluorescence intensity (MFI) of MaMel51, SK‑MEL-28 and WM35 scr and p14^kd^ cells. MFI was normalized to scr control cells of each cell line and significances were determined by unpaired, two-tailed t tests for scr and p14^kd^ cells of each cell line. n=3, mean + SD. p values < 0.05 were considered significant (* for p < 0.05, *** for p < 0.001, **** for p < 0.0001).

## Figure S6
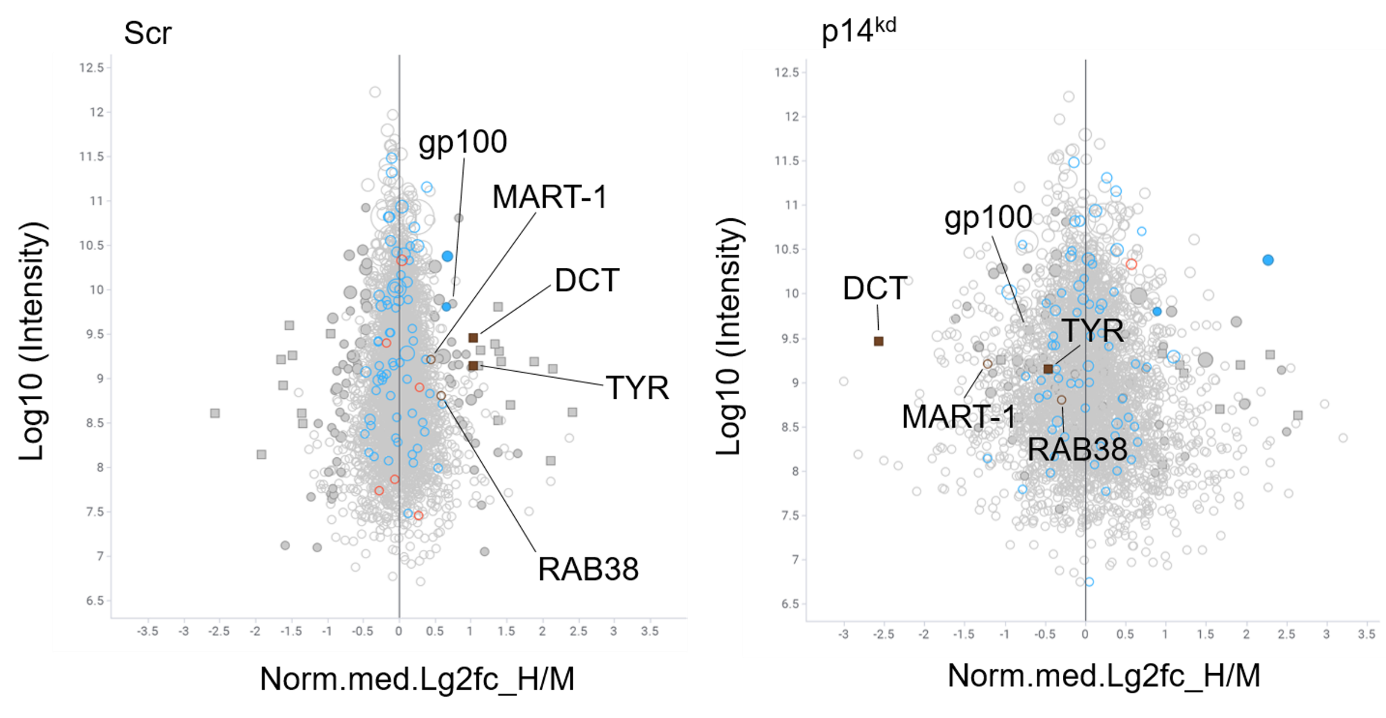


**Figure S6: Melanoma differentiation antigen (MDA) protein expression is decreased upon p14^kd^**.

p14^kd^ induces downregulation of melanoma differentiation antigen (MDA) proteins gp100, MART-1, DCT, TYR and RAB38 in comparison to scr cells. Proteome analysis by liquid chromatography-mass spectrometry (LC-MS) after 48 h of pulsed stable isotope labeling and amino acids in cell culture (pSILAC) treatment of MaMel51 scr and p14^kd^ cells. Normalized median of logarithmic fold changes of specific heavy to medium-labeled proteins in scr control cells and p14^kd^ cells are shown.

## Figure S7
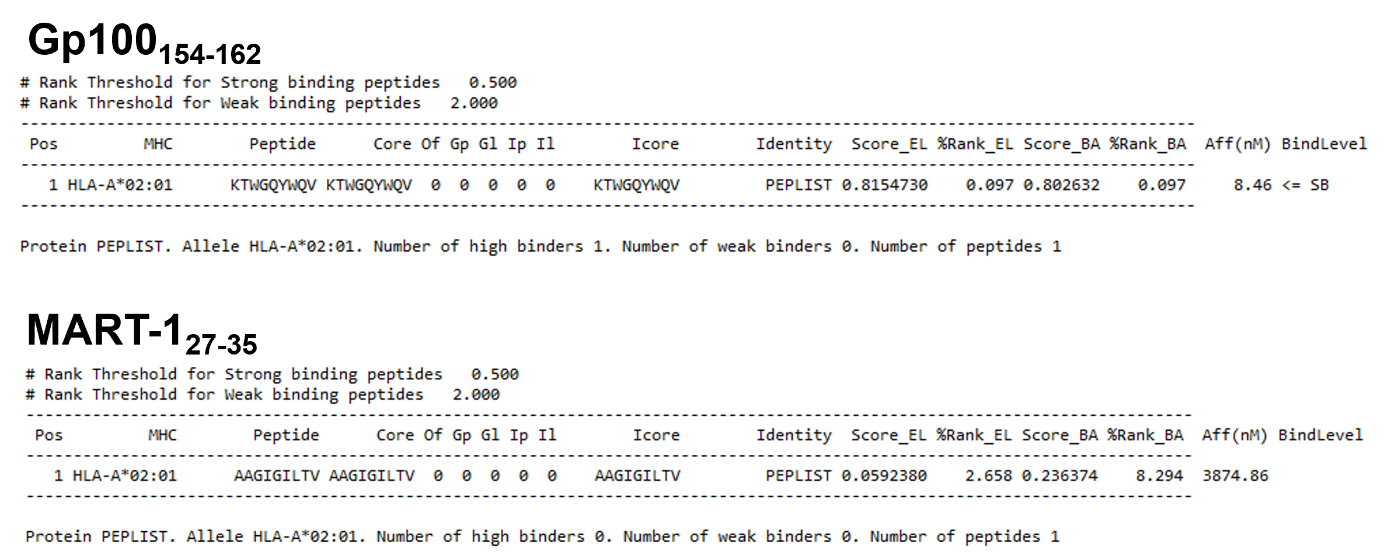


**Figure S7: In silico peptide binding capacity might define binding probability.**

gp100_154-162_ peptide binds stronger to HLA-A*02 compared to MART-1_27-35_ peptide. Excerpt of NetMHCpan 4.1 analysis of binding properties of gp100_154-162_ and MART-1_27-35_ peptides.

## Figure S8


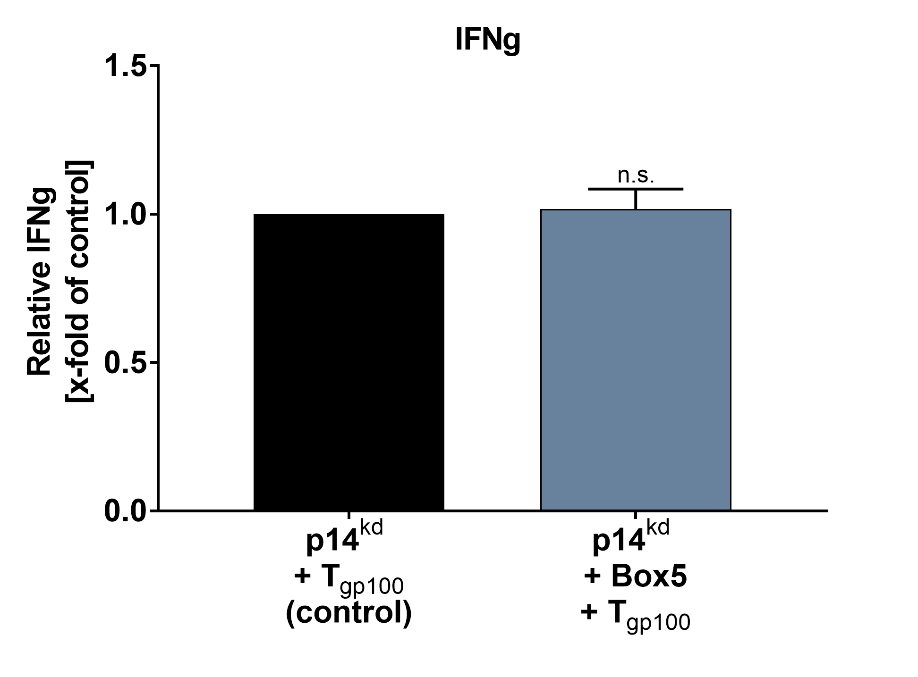


**Figure S8:** **T_gp100_ cell recognition of p14^kd^ cells is not affected by Box5 treatment.**

Measurement of Interferon gamma (IFNg) secretion after coculture of MaMel51 scr or p14^kd^ cells after +- Box5 treatment and T_gp100_ cells. IFNg levels were normalized to p14^kd^ control cells and significance was determined by unpaired, two-tailed t test. n=2, mean + SD. p values < 0.05 were considered significant.
